# Sociodemographic differences in the response to changes in COVID-19 testing guidelines

**DOI:** 10.1093/eurpub/ckae145

**Published:** 2024-10-10

**Authors:** Shambhavi Sharma, Huiqi Li, Jesper Löve, Chioma Nwaru, Magnus Gisslén, Sara Byfors, Niklas Hammar, Anton Nilsson, Jonas Björk, Fredrik Nyberg, Carl Bonander

**Affiliations:** School of Public Health and Community Medicine, Institute of Medicine, University of Gothenburg, Gothenburg, Sweden; School of Public Health and Community Medicine, Institute of Medicine, University of Gothenburg, Gothenburg, Sweden; School of Public Health and Community Medicine, Institute of Medicine, University of Gothenburg, Gothenburg, Sweden; School of Public Health and Community Medicine, Institute of Medicine, University of Gothenburg, Gothenburg, Sweden; Department of Infectious Diseases, Institute of Biomedicine, Sahlgrenska Academy, University of Gothenburg, Gothenburg, Sweden; Region Västra Götaland, Department of Infectious Diseases, Sahlgrenska University Hospital, Gothenburg, Sweden; Public Health Agency of Sweden, Solna, Sweden; Public Health Agency of Sweden, Solna, Sweden; Unit of Epidemiology, Institute of Environmental Medicine, Karolinska Institute, Stockholm, Sweden; Epidemiology, Population Studies and Infrastructures (EPI@LUND), Department of Laboratory Medicine, Lund University, Lund, Sweden; Epidemiology, Population Studies and Infrastructures (EPI@LUND), Department of Laboratory Medicine, Lund University, Lund, Sweden; Clinical Studies Sweden, Forum South, Skåne University Hospital, Lund, Sweden; School of Public Health and Community Medicine, Institute of Medicine, University of Gothenburg, Gothenburg, Sweden; School of Public Health and Community Medicine, Institute of Medicine, University of Gothenburg, Gothenburg, Sweden; Centre for Societal Risk Management, Karlstad University, Karlstad, Sweden

## Abstract

During the coronavirus disease 2019 (COVID-19) pandemic, Sweden emphasized voluntary guidelines over mandates. We exploited a rapid change and reversal of the Public Health Agency of Sweden’s COVID-19 testing guidelines for vaccinated and recently infected individuals as a quasi-experiment to examine sociodemographic differences in the response to changes in pandemic guidelines. We analyzed daily polymerase chain reaction tests from 1 October 2021 to 15 December 2021, for vaccinated or recently infected adults (≥20 years; *n* = 1 596 321) from three Swedish regions (Stockholm, Örebro, and Dalarna). Using interrupted time series analysis, we estimated abrupt changes in testing rates at the two dates when the guidelines were changed (1 November and 22 November). Stratified analysis and meta-regression were employed to explore sociodemographic differences in the strength of the response to the guideline changes. Testing rates declined substantially when guideline against testing of vaccinated and recently infected individuals came into effect on 1 November [testing rate ratio: 0.50 (95% confidence interval, CI 0.41, 0.61)], and increased again from these lowered levels by a similar amount upon its reversal on 22 November [testing rate ratio: 2.19 (95% CI: 1.69, 2.85)]. Being Sweden-born, having higher household income, or higher education, were all associated with a stronger adherent response to the guideline changes. Adjusting for stratum-specific baseline testing rates and test-positivity did not influence the results. Our findings suggest that the population was responsive to the rapid changes in testing guidelines, but with clear sociodemographic differences in the strength of the response.

Key points

- Previous research from the coronavirus disease 2019 (COVID-19) pandemic has documented social disparities in the uptake of preventive measures but there is a lack of data on how populations adapt to rapid changes in non-enforced pandemic guidelines.
- Our study provides quasi-experimental evidence on sociodemographic differences in the response to two rapid, successive changes in COVID-19 testing guidelines for vaccinated and recently infected individuals in Sweden.
- All subgroups studied adjusted their behavior in line with the new guidelines, but the behavioral response was greater among native-born Swedes and individuals with higher socioeconomic status.
- Our results imply that there are strong social differences in the behavioral response to changes in non-enforced guidelines in a pandemic context.

## Introduction

In the global battle against the coronavirus disease 2019 (COVID-19) pandemic, nations faced unprecedented challenges in shaping their policy responses. Due to varying regulations and judgments, each country adopted different approaches. In Sweden, the Communicable Diseases Act emphasizes individual responsibility and voluntary actions to control the spread of infectious diseases, which steered Sweden’s mitigation strategy towards encouraging individuals to voluntarily follow guidelines, in contrast with the enforceable lockdowns implemented in many other countries [1].

A central part of the Swedish strategy involved regularly communicating non-enforced guidelines and advice to the public on reducing infection risks and preventing the spread of the SARS-CoV-2 virus [1]. Similar to health agencies worldwide, the Public Health Agency of Sweden (PHAS) continuously revised its recommendations on social distancing, handwashing, testing, and protocols for infected individuals, following the evolving nature of the pandemic and emerging scientific evidence.

The overall effectiveness of this type of mitigation strategy hinges on the public’s responsivity to changes in pandemic guidelines, which may be influenced by both personality traits and structural barriers (e.g. that not everyone is able to work from home). Social disparities in people’s ability to act according to guidelines raise concerns about negative health inequality impacts [2], and previous research shows clear sociodemographic variations in the uptake of COVID-19 preventive measures as well as in morbidity and mortality [3–5]. However, there is limited data on social disparities in the response to successive changes in pandemic guidelines. To address this gap, we investigated sociodemographic differences in the response to a change in PHAS’s testing guidelines in November 2021 and its subsequent reversal a few weeks later.

## Methods

### Contextual information about the changes in testing guidelines

During most of the COVID-19 pandemic, Sweden’s public healthcare system provided widespread access to community polymerase chain reaction (PCR) testing for the SARS-CoV-2 virus, with the PHAS recommending PCR testing for those suspecting an infection and for contact tracing. However, on 1 November 2021, the PHAS revised its testing guidelines. The revised guidelines specifically noted for fully vaccinated individuals (those who received two doses) and those recently infected (within the past 6 months), “as a rule, this group does not need to get tested for COVID-19” [6].

The rationale for modifying the guidelines was that COVID-19 prevention measures should be no more restrictive than is justified by the threat to human health. With higher vaccination rates lowering the risk of severe illness and transmission, especially among the vaccinated, the PHAS considered it generally unnecessary for fully vaccinated individuals to undergo testing despite showing symptoms. Exceptions to these guidelines included healthcare workers, individuals needing medical care or treatment for COVID-19 symptoms, recipients of home care, nursing home residents, and travelers from non-Nordic countries showing symptoms.

Due to indications of a changing epidemiological situation, the PHAS reversed its stance on 22 November 2021, once again recommending PCR testing for fully vaccinated and recently infected individuals. This change preceded the reporting of the Omicron variant by South Africa on 24 November [7]. Soon after, the first Omicron cases were confirmed in Sweden [8].

Website snapshots of how these testing guidelines were communicated by the PHAS are available in the Supplementary material.

### Data sources

Our study is part of the *Swedish Covid-19 Investigation for Future Insights—A Population Epidemiology Approach using Register Linkage (SCIFI-PEARL)* project, which has a database of individually linked register data for the Swedish population [9], approved for COVID-19 research by the Swedish Ethical Review Authority (2020-01800 with amendments).

As Sweden lacks a national database with both positive and negative PCR tests for its entire population, we relied on PCR test data from three regions (Stockholm, Örebro, and Dalarna) that provided testing data to the COVID-19 quality register [10]. These regions represented 29% of the total Swedish population in 2021 [11]. Sweden’s local self-governance principle gives regions the right to design their testing strategies based on local conditions, though they usually follow the national recommendations by PHAS. During our study period, the three study regions provided various options for self-booking and accessing PCR tests, including unmanned boxes, pharmacies, testing stations, and home delivery services. All three regions communicated the changes in PHAS testing guidelines on their regional websites (snapshots available in the Supplementary material).

To define our study population and carry out stratified analyses, we used data from the *Total Population* Register [12], *SmiNet* [13] (a national disease reporting system containing all positive COVID-19 PCR tests), the *National Vaccination Register* [14] (to identify vaccinated individuals), the *Longitudinal Integrated Database for Health Insurance and Labor Market Studies* [15] (for socioeconomic data and occupational data to identify healthcare workers), and the *Register of Services for the Elderly and People with Disabilities* [16] (to identify people with homecare and nursing home residents).

#### Study population

Our study focused on adults (≥20 years) targeted by the guideline change on 1 November 2021, and its reversal on 22 November 2021. We restricted our study to those residing in the study regions of Stockholm, Örebro, or Dalarna at the pandemic’s onset and remaining in Sweden, alive, at the start of our study period (1 October 2021).

We classified individuals as being targeted by the revised testing guidelines if they had received two vaccine doses for COVID-19 or tested positive for COVID-19 within 6 months before 1 November 2021, excluding healthcare workers, homecare recipients (per October 2021), and nursing home residents (per October 2021). We classified healthcare workers based on occupational data from 2019 (using four-digit Swedish Standard Occupational Classification codes; Supplementary Table S1). Following Kahn *et al.* [17], we also classified additional (suspected) healthcare workers by identifying working-age individuals (<65 years by 1 November 2021) vaccinated with at least one dose before the vaccination program for the general working-age population started (21 April 21 2021, in Stockholm [18], the first of the three included regions to start vaccinating non-healthcare workers below 65 years of age). Applying the above restrictions, 70% of the residents assessed for eligibility in the three regions remained (Supplementary Fig. S1), resulting in a study population of 1 596 321 individuals (79%, 11%, and 10% from Stockholm, Örebro, and Dalarna, respectively).

#### Variables

We assessed our outcome variable in terms of daily per capita PCR tests. To verify the completeness of the outcome data, we compared COVID-19 cases from SmiNet, expected to have all of Sweden’s positive PCR results, with those in the COVID-19 quality register during our study period. We were able to find 81% of the positive cases from SmiNet in the quality register, indicating a satisfactory completeness.

Employing the approach of Kennedy *et al.* [19], we then reviewed and excluded tests reported by patient-treating facilities like hospitals, emergency departments, primary care clinics, and nursing homes, as PHAS’s change in guidelines did not pertain to people who required care for symptoms of COVID-19. For the same reason, we omitted tests from units clearly marked as conducting tests on travelers.

Our exposures of interest were the interventions defined by the change in guidelines on 1 November 2021, and their subsequent reversal on 22 November 2021.

For stratified analyses, we considered the following sociodemographic factors: sex, age (grouped as 20-49, 50-64, 65+ years to approximate low, medium, and high-risk groups for COVID-19 death [20]—65 is also the typical retirement age in Sweden), education level (categorized as primary, secondary, and tertiary), quartile household disposable income based on the regional population assessed for eligibility (Q1–Q4), and birthplace (Sweden or elsewhere). These factors resulted in 144 (2×3×3×4×2) unique intersectional strata [21].

#### Analytical datasets and study period

We compiled time series data by aggregating daily PCR tests from 1 October to 15 December 2021, for the entire study population. We applied the same approach to each sociodemographic stratum. We categorized the study into three periods (“segments”): 1 October–31 2021 (baseline), 1 November–21 2021 (guideline change period), and 22 November–15 December 2021 (reversal period).

### Analysis

**Figure 1.**
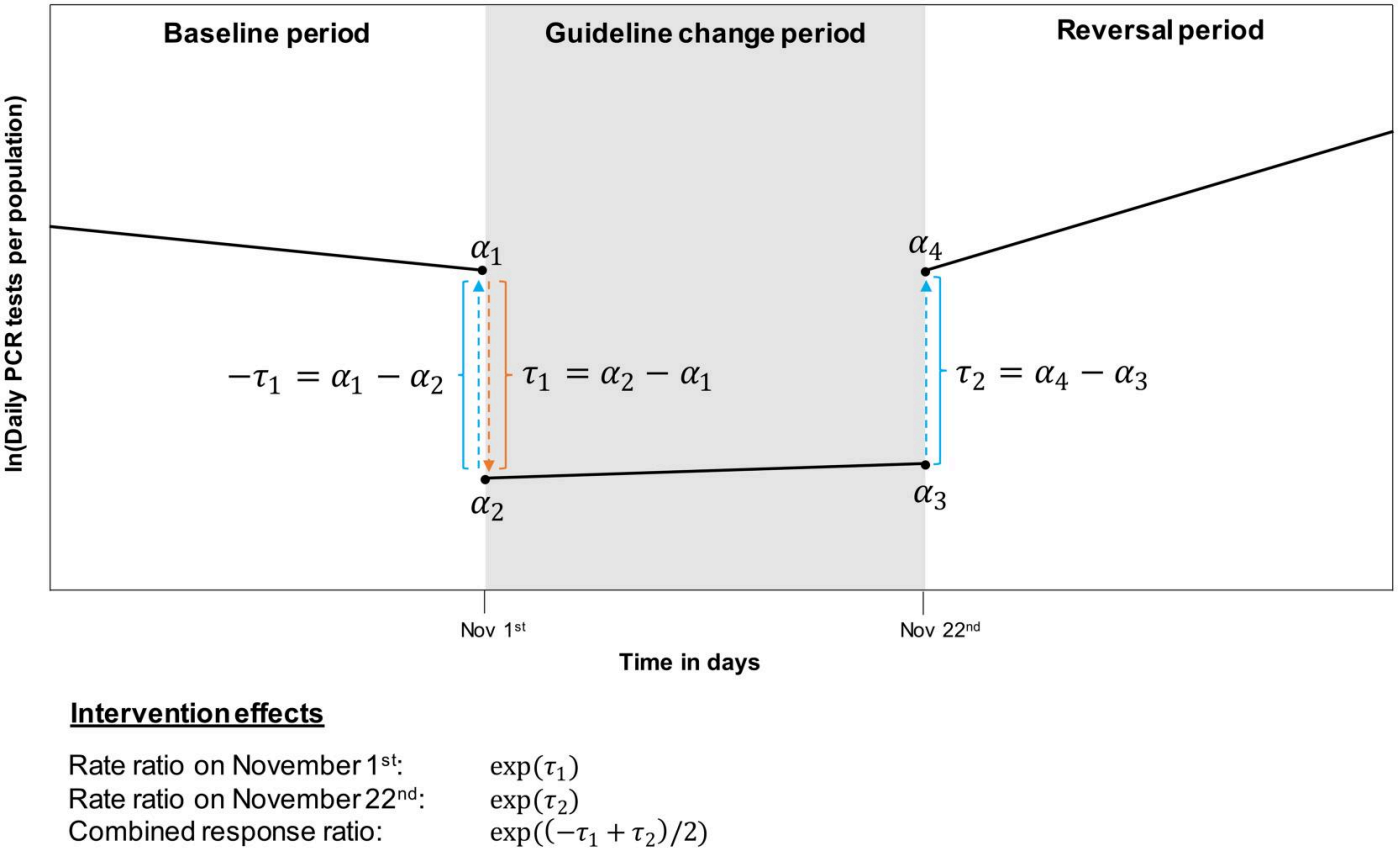
Sketch of the design and modeling strategy used to estimate the impact of the changes in testing guidelines on 1 November 2021 and 22 November 2021, as well as the combined response to both strategies, which involves the inverse effect of the first intervention. Our model allows for different slopes in the three periods, and we focus on the abrupt discontinuities at the intervention dates to estimate their effects.

Figure 1 contains a graphical sketch of the design and modelling strategy (see Supplementary materials for details). Specifically, we specified a segmented Poisson regression model with change points at the two intervention dates, with the daily number of PCR tests as the outcome and population size as an offset to model testing rates per population. Using interrupted time series analysis (ITS) [22], we estimated the impact of each intervention as an abrupt multiplicative change in testing rates on the dates when the guidelines changed, while adjusting for log-linear baseline trend and trend changes across the three segments to account for underlying, gradually changing infection dynamics (e.g. the emergence of the Omicron variant towards the end of our study period). We centered the trend change variables at the first date of each intervention to ensure that the coefficients on the intervention variables quantify the discontinuity in testing rates on 1 November and 22 November, respectively [23, 24]. Our model included a categorical day-of-week variable to adjust for weekday effects, as repetitive patterns (e.g. seasonality) can otherwise cause problems in ITS [22]. To address autocorrelation and other violations of the Poisson modelling assumptions [22, 25], we computed heteroscedasticity and autocorrelation robust standard errors using the “vcovHAC” function from the “sandwich” R package [26], which implements the data-driven estimator by Andrews [27]. We also estimated negative binomial models and linear regressions with and without logged outcomes as sensitivity checks.

Results were presented both with two separate measures (one for each of the two policy changes), and with a single measure, computed as the geometric mean of the two intervention effects. To ensure comparability, we used the inverse effect of the first guideline change in this computation (Fig. 1), resulting in a combined response ratio where a value above one implies that one group, on average across the two interventions, changed their testing behavior in the direction intended by the PHAS (a decrease on 1 November and an increase on 22 November). Standard errors for the combined ratios were obtained using the variance and covariance of the two separate effect estimates.

To enable adjustment for correlated characteristics [28] and identification of within-group variation [29] in the heterogeneity analysis, we computed stratum-specific estimates of the combined response ratio for each of the 144 sociodemographic strata, and their associated standard error on the logarithmic scale. Unadjusted and adjusted associations with the strength of the response were then analyzed using fixed effects inverse variance meta-regressions of the 144 estimates and standard errors [30]. An exponentiated coefficient from these models can be interpreted as the ratio of the combined response ratio between one group and its reference category (i.e. a relative response ratio); if all compared groups respond in the desired direction, a value above one implies a stronger response to the guidelines. In our multivariable specification, we included the main effects of all five of the stratifying variables.

We also performed a *Q*-test to determine if residual variation in effect sizes, unexplained by the main effects, exceeded what would be expected from sampling variability alone, and used $R^{2}$ and $I^{2}$ statistics to assess and estimate the share of the heterogeneity explained by the main effects and the percentage of the residual variability that is due to heterogeneity, respectively [31, 32]. As a sensitivity analysis, we also fit a random-effects meta-regression model to assess the influence of unexplained heterogeneity on the results.

Heterogeneity is scale-dependent [33]. Poisson models estimate multiplicative effects, reflecting our intention to capture proportional behavioral changes across groups while avoiding undue influence of baseline testing rates on the estimates of heterogeneity. In a sensitivity analysis, we also adjusted for each stratum’s baseline testing rate and percent of positive tests (grouped into quartiles) to further address potential biases from differences in baseline testing behaviors, by adding these variables to the meta-regression.

## Results

### Descriptive data

Across the study period, 216 705 PCR tests were recorded in our study population, 179 714 (82.9%) of which were analyzed after excluding tests reported by patient-treating facilities and tests for travelers. During the baseline, the daily average was 93 tests per 100 000 individuals (6.3% of which were positive). In the period when testing was discouraged for vaccinated and recently infected individuals, this number dropped to 62 tests per 100 000 (9.4% positive). In the period after the guideline’s reversal, which also covers the beginning of the Omicron period, the daily count was 294 tests per 100 000 (6.7% positive).

The population’s characteristics are available in Supplementary Table S2. Notably, most individuals (99.1%) were included based on being vaccinated while being recently infected was uncommon (2.4%) (Supplementary Table S2). Compared to the national average, our study population was more likely to live in urban areas and had higher average income and educational attainment (Supplementary Table S3).

### Segmented regression analysis on the entire study population

The segmented regression analysis revealed a decline in testing on 1 November, following the PHAS’s guideline against testing [ratio: 0.50 (95% confidence interval, CI 0.40, 0.61)]. Testing increased again on the guideline’s reversal on 22 November [ratio: 2.19 (95% CI: 1.69, 2.85)], resulting in a combined response ratio of 2.10 (95% CI: 1.88, 2.34).

**Figure 2.**
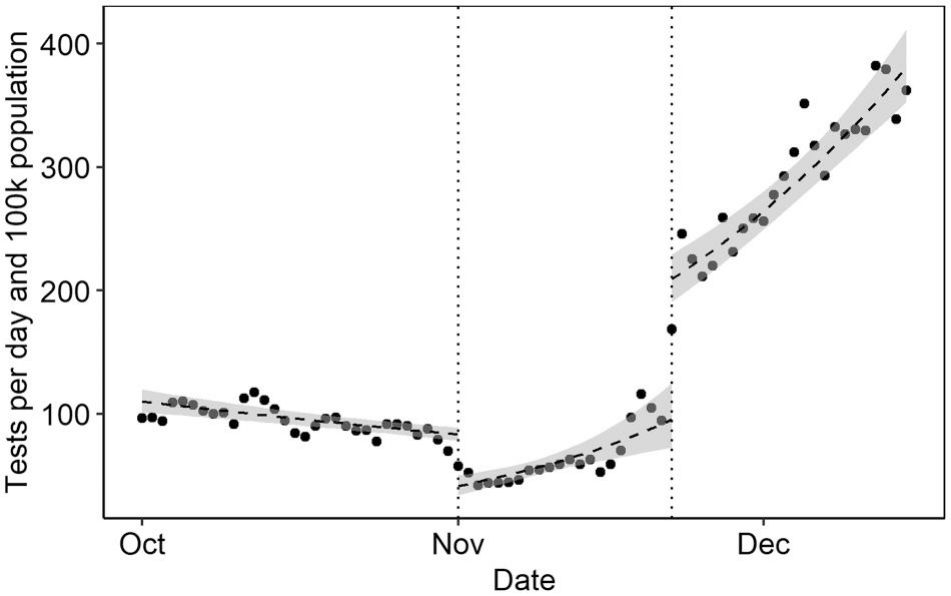
Daily counts of PCR tests per 100 000 population in the study population of individuals vaccinated for or infected by COVID-19 within the last 6 months from three regions in Sweden (Stockholm, Örebro, and Dalarna) from 1 October to 15 December 2021, with segment-specific log-linear trend lines from the segmented regression model. Points represent observed data after controlling for day of week effects based on the day of week coefficients in the segmented regression model (for a non-residualized version of the data points, see Supplementary Fig. S1). The first vertical line marks the beginning of the no-testing guideline on 1 November, and the second the reversal of this guideline change on 22 November.

The daily testing rates, with day-of-week effects removed, are visualized in Fig. 2 together with model-based time trends (Supplementary Fig. S2 shows the same data including day-of-week effects). The results were consistent with alternative regression models (Supplementary Table S4).

### Region-specific subgroup analyses

There were discontinuities in testing on both intervention dates in all three regions, although the average response was greater in Stockholm [combined response ratio: 2.21 (95% CI: 1.94, 2.53)] and Dalarna [2.58 (95% CI: 2.20, 3.03)] than in Örebro [1.68 (95% CI: 1.50, 1.87)] (Supplementary Table S5).

### Sociodemographic effect heterogeneity

All subgroups showed a shift in their testing behavior in the expected direction following the two guideline changes (reduction in testing on 1 November, increase on 22 November), but with substantial variations in the proportional change across groups (Table 1).

**Table 1.** Estimated impact of the two recommendation changes issued by the Public Health Agency of Sweden in November 2021, on daily testing rates in the overall study population of individuals vaccinated for or infected by COVID-19 within the last 6 months from three regions in Sweden (Stockholm, Örebro, and Dalarna) and sociodemographic subgroups

	Daily tests per 100 000 population			Rate ratio estimates for abrupt changes in testing rates at recommendation change dates (95% CI)		
Group	1 October–31 October	1 November–21 November	22 November–15 December	1 November	22 November	Combined response ratio^a^
Study population	93	62	294	0.50 (0.41, 0.61)	2.19 (1.69, 2.85)	2.10 (1.88, 2.34)
Sex						
Female	114	74	341	0.49 (0.40, 0.59)	2.14 (1.67, 2.74)	2.10 (1.89, 2.33)
Male	74	50	251	0.51 (0.40, 0.65)	2.27 (1.66, 3.11)	2.11 (1.86, 2.39)
Age group						
20–49 years	127	83	386	0.51 (0.41, 0.62)	2.12 (1.60, 2.79)	2.05 (1.83, 2.29)
50–64 years	87	58	306	0.44 (0.33, 0.59)	2.32 (1.63, 3.30)	2.29 (1.97, 2.66)
65+ years	36	26	111	0.57 (0.37, 0.87)	2.34 (1.46, 3.76)	2.03 (1.65, 2.49)
Education						
Primary	55	36	152	0.58 (0.48, 0.69)	1.95 (1.42, 2.68)	1.84 (1.63, 2.07)
Secondary	86	56	267	0.49 (0.39, 0.60)	2.08 (1.64, 2.65)	2.07 (1.87, 2.29)
Tertiary	111	74	360	0.49 (0.38, 0.64)	2.30 (1.67, 3.15)	2.16 (1.89, 2.47)
Family income						
Q1 (lowest)	80	51	199	0.57 (0.47, 0.70)	1.92 (1.52, 2.42)	1.83 (1.66, 2.02)
Q2	107	70	317	0.50 (0.41, 0.60)	1.94 (1.47, 2.55)	1.98 (1.77, 2.21)
Q3	106	69	345	0.48 (0.37, 0.62)	2.29 (1.65, 3.18)	2.19 (1.92, 2.50)
Q4 (highest)	79	55	284	0.48 (0.36, 0.64)	2.46 (1.75, 3.47)	2.26 (1.96, 2.62)
Country of birth						
Sweden	99	63	320	0.46 (0.37, 0.56)	2.24 (1.72, 2.92)	2.22 (1.98, 2.48)
Elsewhere	73	57	200	0.71 (0.61, 0.84)	1.88 (1.52, 2.34)	1.62 (1.48, 1.78)

a Geometric mean of the two recommendation changes, with the coefficient on 1 November inverted (see text for details). A value above 1 implies a response in the expected direction.

**Figure 3.**
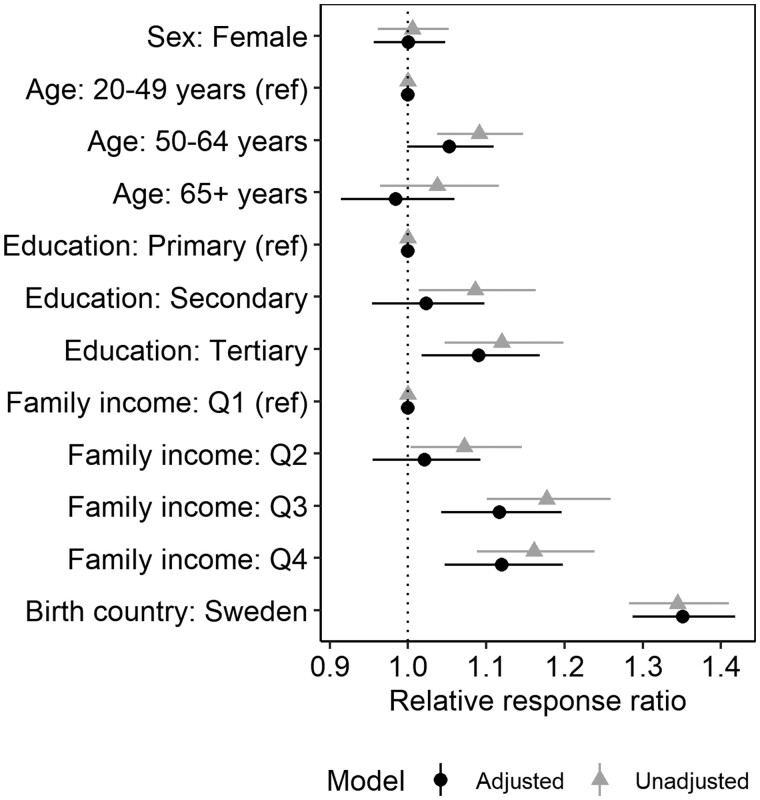
Results from the effect heterogeneity analysis, showing adjusted and unadjusted estimates of sociodemographic differences in the relative size of the response ratio estimating the combined effect of the two changes testing guidelines in the study population of individuals vaccinated for or infected by COVID-19 within the last 6 months from three regions in Sweden (Stockholm, Örebro, and Dalarna; period: 1 October to 15 December 2021). A value above 1 indicates a stronger adherent response in a group compared to its reference group, marked with (ref) for multi-category variables. For sex, males are the reference group, and for birth country, “elsewhere” is the reference.

Figure 3 contains a forest plot with unadjusted and adjusted coefficients from the meta-regression analysis. Overall, the results indicate that being born in Sweden, having higher family income, and having advanced education were associated with stronger adherent responses (Fig. 3; estimates from all 144 strata are also shown in Supplementary Fig. S3). Age and sex were less consistently associated with relative changes in testing across unadjusted and adjusted models.

The test for residual heterogeneity suggests that the main effects jointly explain 48.8% of the variation in the stratum-specific effect estimates with some residual heterogeneity left unexplained (*I*^2^ = 23.4%, *P* = 0.001).

Similar results were obtained when adjusting for differences in baseline testing rates and baseline test positivity at the group level and using a random-effects specification for the meta-regression (Supplementary Figs S4–S5).

## Discussion

Our study used a unique quasi-experiment in Sweden to explore the public’s behavioral response to rapid changes in pandemic guidelines. We observed significant shifts in testing rates coinciding with the changes in PHAS’s testing guidelines: a decrease following the initial change and an increase after its reversal. These shifts, which were both in the expected direction, suggest an overall responsiveness to pandemic guidelines in our study population. This result is in line with research on other pandemic interventions in the country, such as an age-specific stay-at-home recommendation for older adults [34].

We also found notable variations in behavioral response to the rapid changes in guidelines across socioeconomic groups. Sweden-born people, individuals with higher family income, and those with advanced education showed a greater response to the rapid changes in testing recommendations. This trend is consistent with previous research on COVID-19 protective behaviors in general [3] and vaccination uptake [35].

Our study cannot answer why different groups responded differently to the changes in testing recommendations or how to increase guideline responsiveness in groups with low adherence. Several factors, including access to information, institutional trust, structural barriers (e.g. poor job security), and perceived susceptibility likely contribute to the observed disparities by impacting both the willingness and ability to follow changing health guidelines [3, 36–39]. Increasing guideline adherence is a non-trivial task, and will likely require a combination of communication strategies and creating appropriative incentives to follow guidelines across sociodemographic groups. Future research should aim to gather more detailed data on attitudes, perceptions, and the specific barriers faced by different groups to inform these strategies. The presence of residual heterogeneity within the broad sociodemographic groups in our model also suggests that it may be appropriate to adopt an intersectional approach in future work [21].

It is noteworthy that within most sociodemographic subgroups, the magnitude of the reductions and increases in testing rates at the two guideline changes were remarkably similar; generally, in groups testing rates halved following the no-testing guideline, they roughly doubled from this reduced rate upon its reversal (Table 1). This pattern suggests that the public’s attentiveness to guideline changes remained relatively consistent, despite the quick reversal which one could speculate would contribute to a lessened trust and attentiveness to the guidelines put out by the PHAS. However, the rising infection pressures in the late study period may also have heightened the public’s attentiveness at the time when the guideline was reversed.

Our study’s primary strength lies in its quasi-experimental design, which accounts for unobserved confounders that evolve smoothly over time (e.g. changing infection pressures) [22]. Our use of comprehensive public and semi-public health and sociodemographic data from a country with universal healthcare reduces the risk of reporting and non-response biases. While the testing data used may not be fully complete, our findings should remain robust, provided there were no abrupt changes in data quality at the intervention dates. Additionally, Sweden’s unique personal identification numbers and register infrastructure enabled us to stratify our analyses using individual-level sociodemographics. However, our study was also limited by the absence of rapid at-home antigen test data. Data on recent infections according to rapid home tests would have enabled us to classify non-targeted individuals more properly and study if and how they also responded to the guideline changes.

While Sweden’s guideline-driven pandemic response is an interesting case, our results may not be directly transferable to other settings. For example, the high level of institutional trust typically seen in the Swedish population could amplify the observed responses to guideline changes compared to places with lower institutional trust [40]. Moreover, responses to other types of guidelines, like social distancing, may not mirror the sociodemographic patterns seen here. Our study also predominantly involved vaccinated individuals, who may naturally be more responsive to guideline changes than unvaccinated people. Hence, the overall responsiveness in our study could be higher than in populations with a larger share of unvaccinated individuals. Our sample also consisted primarily of individuals from Stockholm, which has a predominantly urban population with higher-than-average income levels and educational attainment, which should be considered when attempting to generalize our findings.

Our analytical approach also has limitations, such as its focus on immediate effects at intervention dates, while not considering time-varying effects or potential anticipation behaviors. Its validity depends on the assumption that no other events influencing testing rates occurred simultaneously with the interventions. A potential concern is the overlap of the first guideline change with a national school break (1 November to 7 November 2021). However, this one-week break cannot account for the subsequent increase in testing on 22 November.

In conclusion, our study indicates that although the Swedish population generally adjusted to the rapid changes in testing guidelines, response varied substantially between different sociodemographic groups, with native-born and socioeconomically advantaged people responding stronger. While each context is unique, it is not unreasonable to assume that somewhat similar patterns may be observed in the future and elsewhere, meaning that our results can be useful for foreseeing some of the consequences of other introductions or abolitions of health recommendations.

## Supplementary Material

ckae145_Supplementary_Data

## Data Availability

The data in this study are pseudonymized individual-level data from Swedish registers and can be obtained from the respective Swedish public data holders on the basis of ethics approval for the research in question, subject to relevant legislation, processes, and data protection.
