# Supplementary material for: Sociodemographic differences in the response to changes in COVID-19 testing guidelines
Source: Eur J Public Health. 2024 Oct 10;34(6):1066–72. doi: 10.1093/eurpub/ckae145 (PMC11631532; doi:10.1093/eurpub/ckae145)
Supplement: ckae145_Supplementary_Data [file ckae145_supplementary_data.pdf]

# Supplement for “Sociodemographic differences in the response to changes in COVID-19 testing guidelines”

**Contents**


| | |
|---|---|
| Figure S1 | Page 2 |
| Figure S2 | Page 3 |
| Figure S3 | Page 4 |
| Figure S4 | Page 5 |
| Figure S5 | Page 6 |
| Table S1 | Page 7 |
| Table S2 | Page 8 |
| Table S3 | Page 9 |
| Table S4 | Page 10 |
| Table S5 | Page 11 |
| Appendix S1 | Page 12 |
| Appendix S2 | Page 14 |

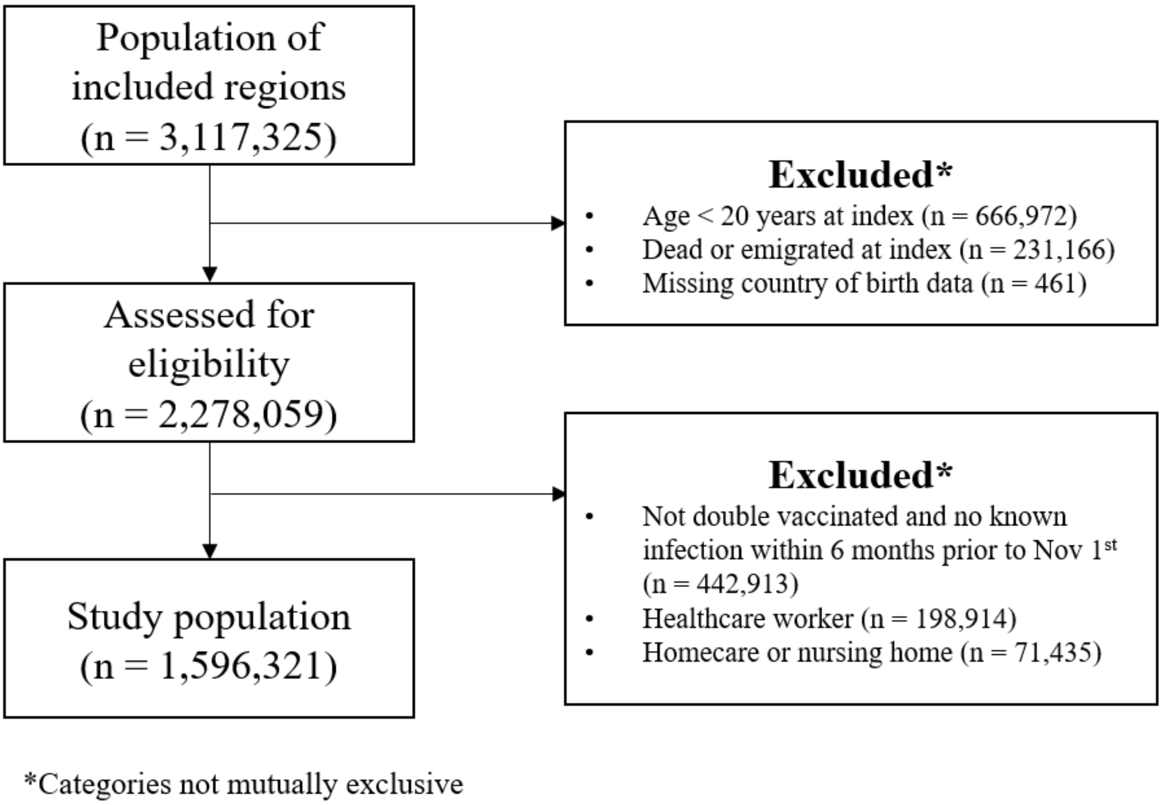


**Figure S1.** Flow chart of the selection process for the study population of individuals vaccinated for or infected by COVID-19 within the last six months from three regions in Sweden (Stockholm, Örebro, and Dalarna).

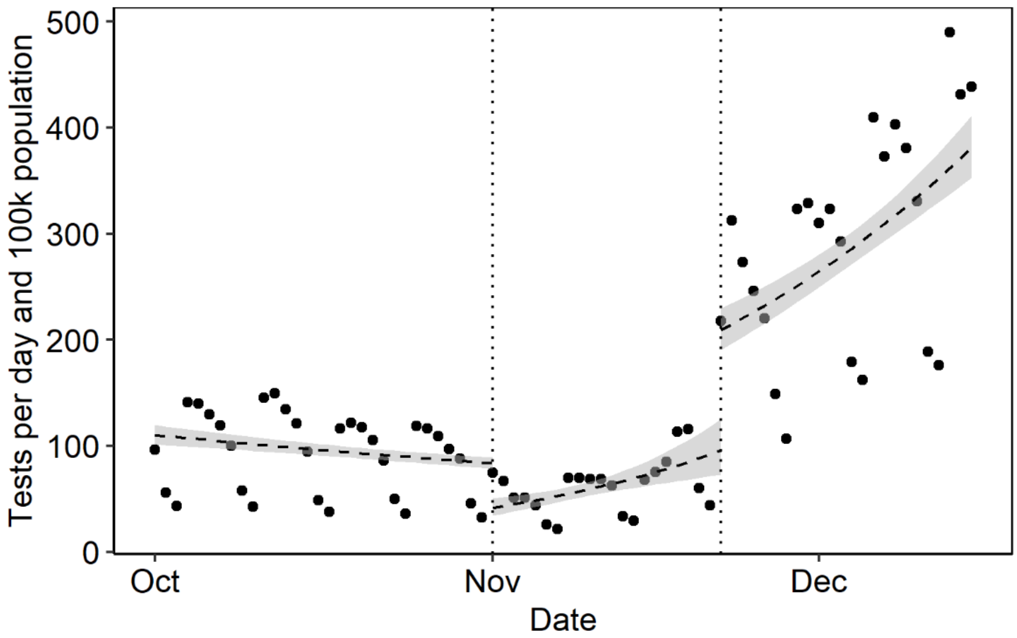


**Figure S2.** Daily counts of patient-initiated PCR tests per 100 000 population in the study population with segment-specific trend lines from the segmented regression model (non-residualized version).

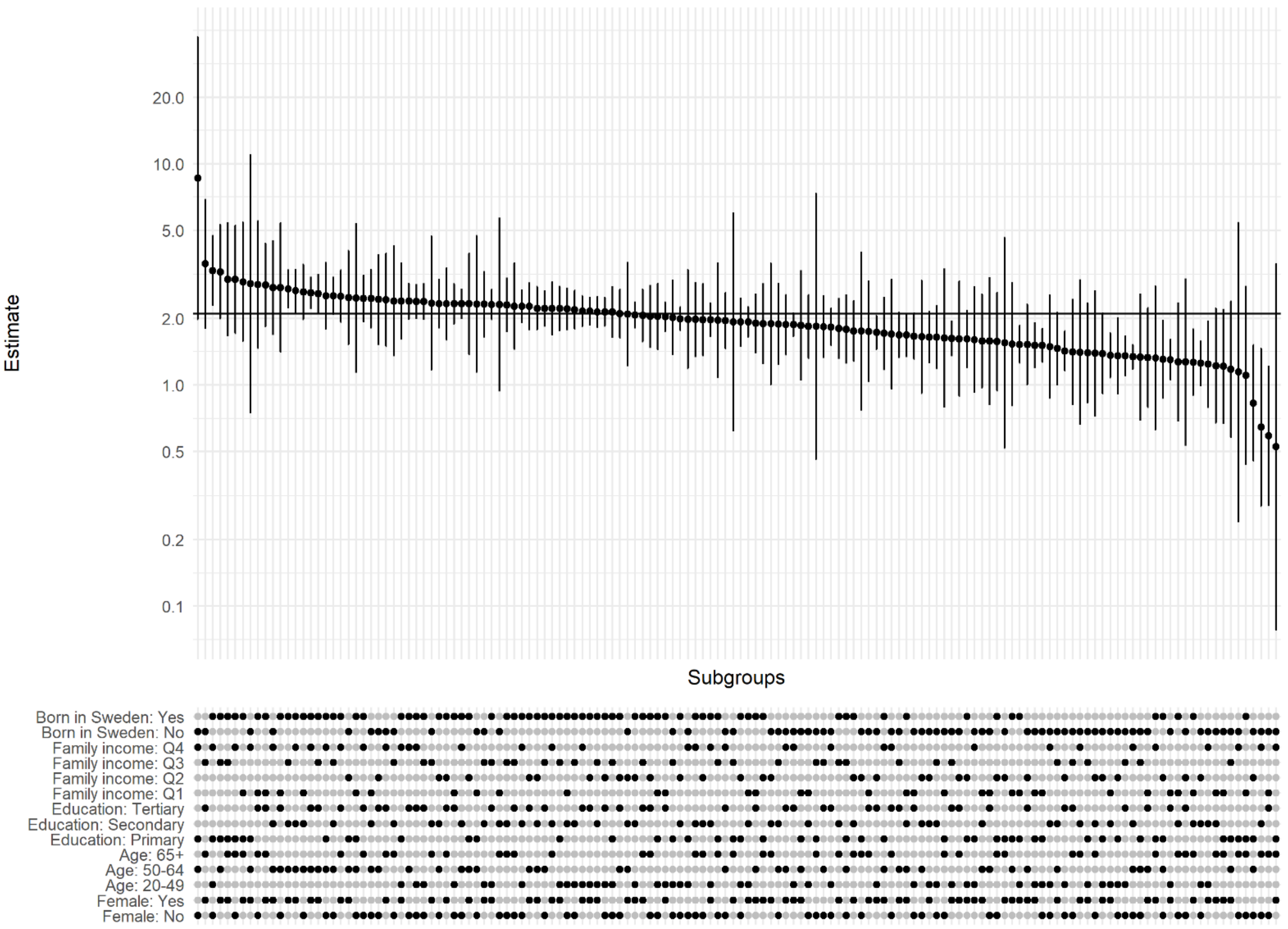


**Figure S3.** Stratum-specific estimates of the combined response ratio to the two guideline changes. In the upper panel, dots represent point estimates and lines 95% confidence intervals. In the bottom panel, a black dot means that the category is included in the stratum and a gray dot means that it is not. Horizontal reference line shows the overall response ratio in the study population (i.e., the mean effect estimate) for reference.

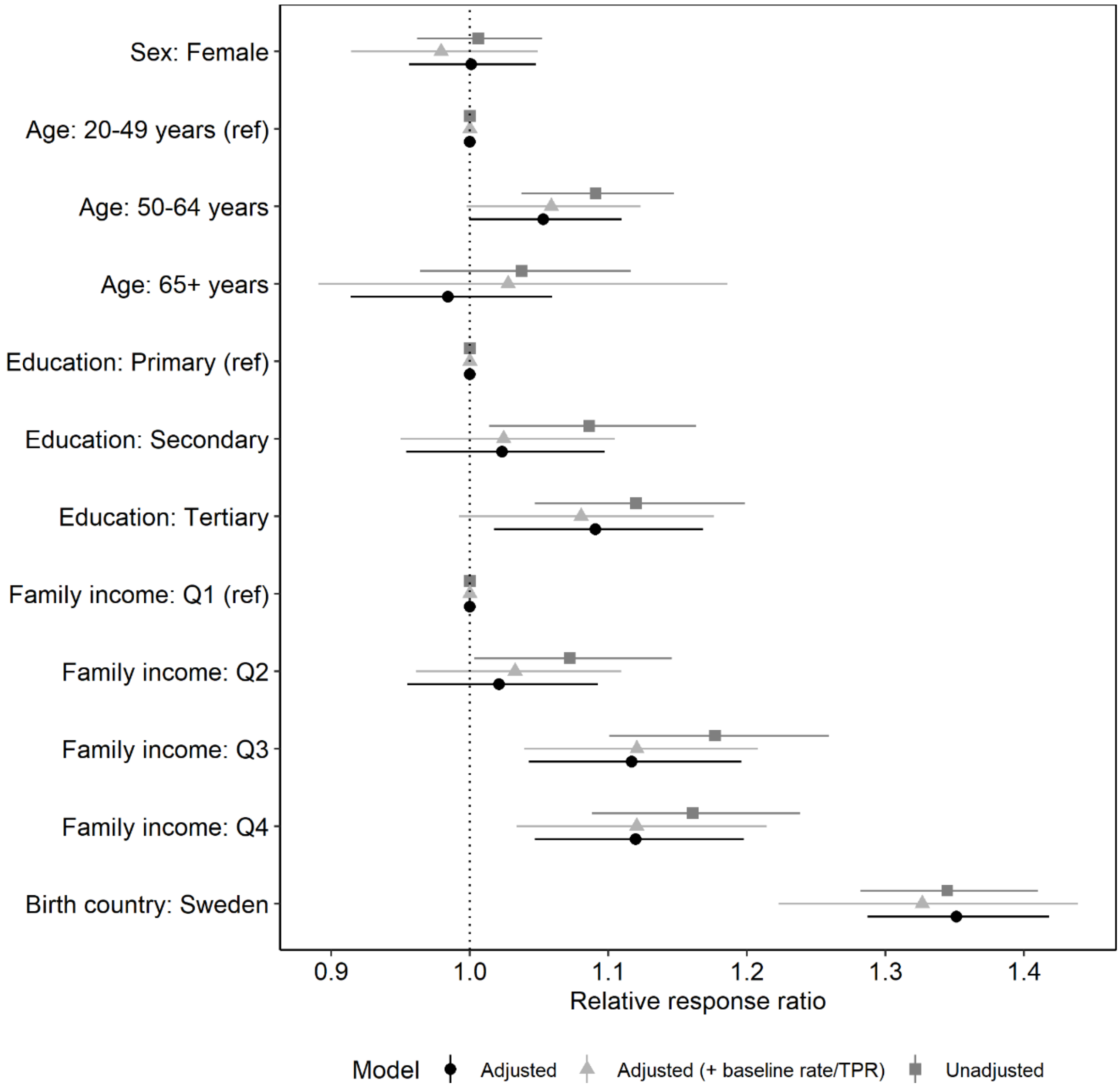


**Figure S4.** Results from the effect heterogeneity analysis, including an additional analysis adjusting for stratum-level differences in baseline testing rates per population and baseline test-positivity rate (TPR; percent of performed tests that were positive), both categorized into quartile groups. A value above one indicates a stronger response in the expected direction in a group compared to their reference group.

.

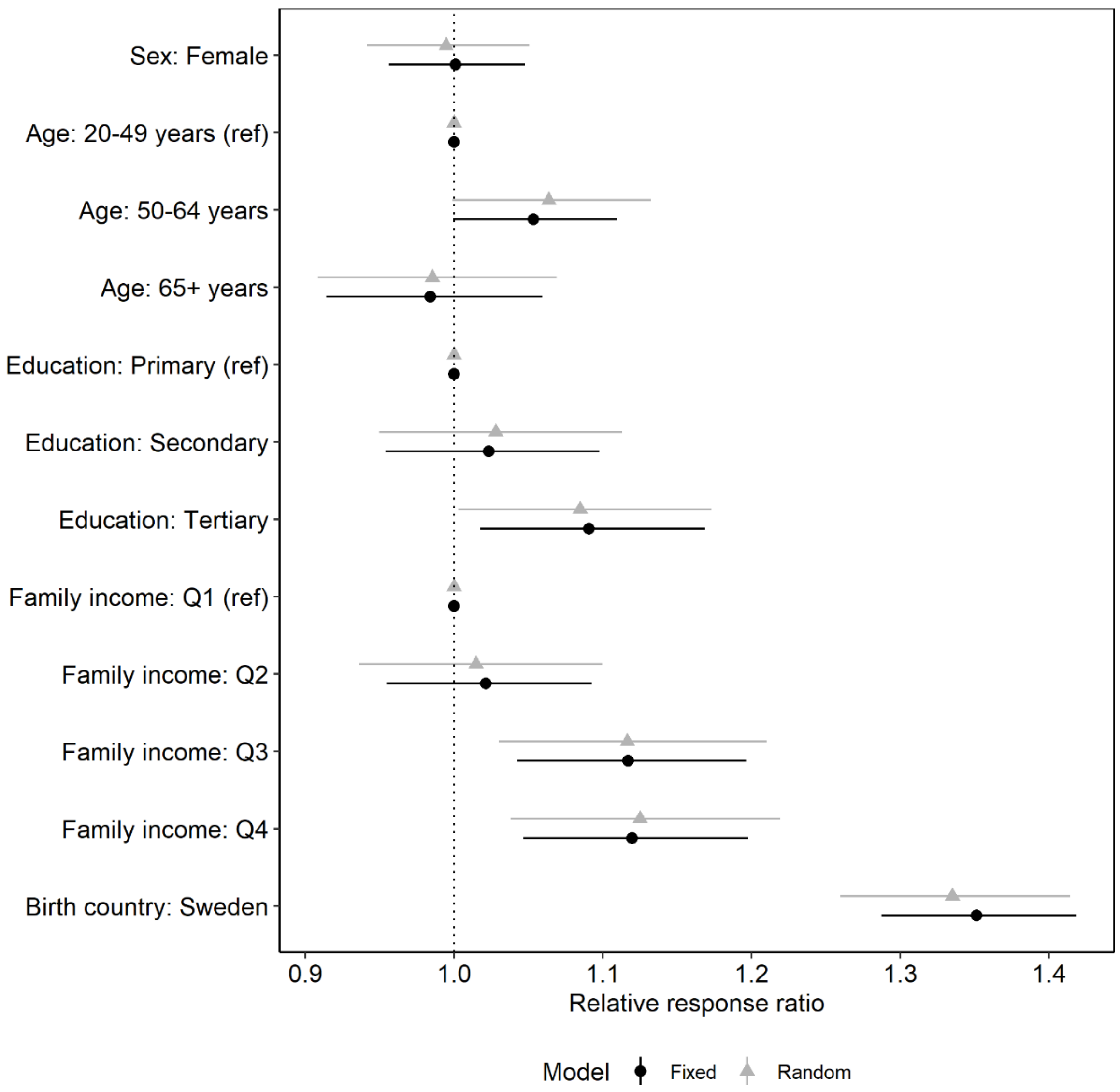


**Figure S5.** Comparison of adjusted effect heterogeneity results from fixed and random effects meta-regression models. A value above one indicates a stronger response in the expected direction in a group compared to their reference group.

**Table S1.** Identifications of occupations classified as 'healthcare workers' in our study using The Swedish Standard Classification of Occupations 2012 (SSYK12) codes.

| SSYK12 | Occupations |
|---|---|
| 2211 | Specialist physicians |
| 2212 | Resident physicians |
| 2213 | General practitioners |
| 2219 | Other physicians |
| 2260 | Dentist |
| 2221 | Professional nurses |
| 2222 | Professional midwives |
| 2223 | Anesthesia nurses |
| 2224 | District nurses |
| 2226 | Nurses- ambulance |
| 2227 | Nurses-geriatric |
| 2228 | Nurses- intensive care |
| 2231 | Nurses-operation |
| 2232 | Nurses-children |
| 2235 | Nurses-radiology |
| 2239 | Other specialist nurses |
| 2271 | Chiropractors and naprapaths |
| 2272 | Physiotherapists |
| 2273 | Occupational therapist |
| 2289 | Health professional not elsewhere classified |
| 3250 | Dental hygienists |
| 5350 | Dental nurses |
| 2284 | Opticians |
| 5321 | Assistant nurses, homecare and homes for the elderly |
| 5322 | Assistant nurses, rehabilitation |
| 5323 | Assistant nurses, hospital ward |
| 5324 | Assistant nurses, clinic |
| 5325 | Assistant nurses, children |
| 5326 | Ambulance attendants |
| 5330 | Home-based personal care and related workers |

**Table S2.** Characteristics of the study population of individuals vaccinated for or infected by COVID-19 within the last six months from 3 regions in Sweden (Stockholm, Örebro, and Dalarna), October 1st to December 15th, 2021.

| Characteristic | Study population (n = 1,596,321) |
|---|---|
| Female, n (%) | 772,105 (48.4) |
| Age group, n (%) | |
| 20-49 years | 780,714 (48.9) |
| 50-64 years | 400,011 (25.1) |
| 65+ years | 415,596 (26.0) |
| Educational attainment, n (%) | |
| Primary or unknown | 226,876 (14.2) |
| Secondary | 617,910 (38.7) |
| Tertiary | 751,535 (47.1) |
| Family disposable income, n (%) | |
| Q1 (lowest) | 271,235 (17.0) |
| Q2 | 383,192 (24.0) |
| Q3 | 448,092 (28.1) |
| Q4 (highest) | 493,802 (30.9) |
| Born in Sweden, n (%) | 1,256,956 (78.7) |
| Vaccinated (two doses)[a], n (%) | 1,582,013 (99.1) |
| Confirmed infection within six months[a], n (%) | 38,185 (2.4) |

a By November 1st, 2021

**Table S3.** Sociodemographic comparison of the full study population and subpopulations from the three study regions (Stockholm, Örebro, Dalarna), with the Swedish national population aged ≥20 years and alive and living in Sweden by October 1st, 2021.

| | | *Study population* | | | |
|---|---|---|---|---|---|
| | Sweden | Full | Stockholm | Örebro | Dalarna |
| N | 8,083,249 | 1,596,321 | 1,263,845 | 170,715 | 161,761 |
| Female, n (%) | 4,044,357 (50.0) | 772,105 (48.4) | 617,862 (48.9) | 80,016 (46.9) | 74,227 (45.9) |
| Age group, n (%) | | | | | |
| 20-49 years | 4,071,181 (50.4) | 780,714 (48.9) | 639,312 (50.6) | 77,088 (45.2) | 64,314 (39.8) |
| 50-64 years | 1,896,576 (23.5) | 400,011 (25.1) | 319,879 (25.3) | 40,249 (23.6) | 39,883 (24.7) |
| 65+ years | 2,115,492 (26.2) | 415,596 (26.0) | 304,654 (24.1) | 53,378 (31.3) | 57,564 (35.6) |
| Educational attainment, n (%) | | | | | |
| Primary or unknown | 1,688,453 (20.9) | 226,876 (14.2) | 163,753 (13.0) | 31,422 (18.4) | 31,701 (19.6) |
| Secondary | 3,434,165 (42.5) | 617,910 (38.7) | 454,488 (36.0) | 81,252 (47.6) | 82,170 (50.8) |
| Tertiary | 2,960,631 (36.6) | 751,535 (47.1) | 645,604 (51.1) | 58,041 (34.0) | 47,890 (29.6) |
| Family disposable income[a], n (%) | | | | | |
| Q1 (lowest) | 1,972,110 (25.0) | 268,930 (16.8) | 193,079 (15.3) | 38,999 (22.8) | 36,852 (22.8) |
| Q2 | 1,972,110 (25.0) | 334,348 (20.9) | 238,981 (18.9) | 48,416 (28.4) | 46,951 (29.0) |
| Q3 | 1,972,109 (25.0) | 411,761 (25.8) | 322,713 (25.5) | 45,955 (26.9) | 43,093 (26.6) |
| Q4 (highest) | 1,972,109 (25.0) | 581,282 (36.4) | 509,072 (40.3) | 37,345 (21.9) | 34,865 (21.6) |
| Born in Sweden, n (%) | 6,186,756 (76.5) | 1,256,956 (78.7) | 967,393 (76.5) | 145,390 (85.2) | 144,173 (89.1) |
| Rural[b], n (%) | 1,237,917 (15.3) | 125,732 (7.9) | 51,740 (4.1) | 33,995 (19.9) | 39,997 (24.7) |

[a] Quantiles defined in relation to the Swedish national population for the purpose of this comparison.

[b] Place of residence was determined using Demographic Statistical Areas (DeSOs, according to the Swedish acronym). Rural areas were classified as those not situated within towns or cities, identified by the presence of the letter 'A' in the fifth position of the DeSO code.

**Table S4.** Segmented regression results with alternative model specifications.

| | Effect estimate (95% CI) | | |
|---|---|---|---|
| Specification | *November 1st* | *November 22nd* | *Combined response* |
| Negative binomial (RR) | 0.50 (0.39, 0.64) | 2.15 (1.58, 2.94) | 2.07 (1.81, 2.37) |
| Logged linear regression (RR) | 0.50 (0.40, 0.63) | 2.18 (1.59, 2.99) | 2.09 (1.84, 2.38) |
| Linear regression (RD) | -63.2 (-97.7, -28.7) | 98.1 (42.5, 153.7) | 80.6 (55.0, 106.2) |

*Notes:* RR = Rate ratio, RD = Rate difference per 100.000 population. All confidence intervals (CIs) are based on heteroscedasticity and autocorrelation robust covariance matrix estimation.

**Table S5.** Segmented regression results by region.

| | Rate ratio (95% CI) | | |
|---|---|---|---|
| Region | *November 1st* | *November 22nd* | *Combined response* |
| Stockholm | 0.52 (0.40, 0.67) | 2.55 (1.85, 3.51) | 2.21 (1.94, 2.53) |
| Örebro | 0.55 (0.48, 0.64) | 1.54 (1.22, 1.96) | 1.68 (1.50, 1.87) |
| Dalarna | 0.35 (0.24, 0.52) | 2.36 (1.58, 3.52) | 2.58 (2.20, 3.03) |

# Appendix S1: Model details

Our segmented Poisson regression model can be expressed as follows:

$$\ln\frac{Y_t}{P} = \alpha + \tau_1 Nov1_t + \tau_2 Nov22_t + \beta_1 Time_t + \beta_2\big(Nov1_t \times (Time_t - t_1)\big)$$
$$+ \beta_3\big(Nov22_t \times (Time_t - t_2)\big) + \sum_{j=1}^{6} \delta_j Weekday_{jt}.$$

In this model, $Y_t$ represents the number of PCR tests on day $t$, and $P$ is the population size, functioning as an offset to quantify rates per population and assumed constant throughout the period. The intervention dummies, $Nov1_t$ and $Nov22_t$, are coded as one after November 1st and November 22nd, respectively. $Time_t$ marks the progression from the study's start, while $t_1$ and $t_2$ denotes the first day of the first and second interventions, respectively. Finally, $Weekday_{jt}$ represents a dummy for weekday $j$, which is included to adjust for day of week-effects (with one dummy, $j$ = *7*, omitted as a reference category). With this specification, where the interacted time variables are centered at $t_1$ and $t_2$,[1] the coefficients $\tau_1$ and $\tau_2$ estimate the abrupt discontinuity in testing rate at the start of the two interventions. The remaining coefficients represent log-linear time trends ($\beta_1$) and log-linear deviations from these trends in the different time segments ($\beta_2$ and $\beta_3$), which serve the purpose of adjusting for underlying, gradually changing infection dynamics (e.g., the emergence of the Omicron variant towards the end of our study period).[2] Causal identification, akin to a temporal regression discontinuity design[3], relies on the assumption that nothing else changed abruptly on November 1st and November 22nd that can explain observed discontinuities in testing rates[4].

Meta-regression models were estimated using the logged combined response ratio (see main text for details) and its standard error from segmented Poisson regression models fit to each of the 144 intersectional strata. Our meta-regression specification contained the main effects of each of the stratum variables as potential moderating variables:

$$\gamma_s = \nu_0 + \nu_1 Female_s + \nu_2 Age5064_s + \nu_3 Age65_s + \nu_4 EducSecondary_s + \nu_4 EducTertiary_s$$
$$+ \nu_5 FamIncQ2_s + \nu_6 FamIncQ3_s + \nu_7 FamIncQ4_s + \nu_8 BornSweden_s + e_s$$

where $\gamma_S$ is the stratum-specific logged response ratio and $e_S$ is a residual error term containing noise and heterogeneity that is not explained by the main effects.

## References


1. Xiao H, Augusto O, Wagenaar BH. Reflection on modern methods: a common error in the segmented regression parameterization of interrupted time-series analyses. *Int J Epidemiol*. 2021 Jun 1;**50**(3):1011–1015.

2. Wagner AK, Soumerai SB, Zhang F, Ross-Degnan D. Segmented regression analysis of interrupted time series studies in medication use research. *J Clin Pharm Ther*. 2002;**27**(4):299–309.

3. Hilton Boon M, Craig P, Thomson H, Campbell M, Moore L. Regression Discontinuity Designs in Health. *Epidemiol Camb Mass*. 2021 Jan;**32**(1):87–93.

4. Hahn J, Todd P, Van der Klaauw W. Identification and Estimation of Treatment Effects with a Regression-Discontinuity Design. *Econometrica*. [Wiley, Econometric Society]; 2001;**69**(1):201–209.

# Appendix S2: Website snapshots

The following appendix contains snapshots of the Public Health Agency's website obtained via the Internet Archive's "Wayback Machine" (https://archive.org/web/), which saves snapshots of websites on specific dates, especially when there is heavy traffic. We extracted communication about the two recommendation changes from the Public Health Agency website (www.folkhalsomyndigheten.se) from the recommendation change dates (or nearby dates), and communication about how to order tests from the regional websites (www.regionstockholm.se; www.regiondalarna.se; www.regionorebrolan.se). We accessed and saved the information in English whenever possible, but some of the saved information below is presented in Swedish form.

The Wayback Machine - https://web.archive.org/web/20211024125857/https://www.folkhalsomyndigheten.se/the-public-health-agency-of-sweden/com…



# What applies from 1 November

**Here you will find information about the COVID-19 testing guidelines and the advice to stay home that will apply as of 1 November.**

On 1 November, changes will be made to the advice for children and adults to stay home if they have symptoms.

Changes will also be made to the testing guidelines.

The guidelines aim to limit the spread of COVID-19 as well as other respiratory tract infections such as the respiratory syncytial virus (RSV) and influenza. They will also protect those most at risk of becoming seriously ill.

The changes introduced on 29 September will still apply. On 29 September, restrictions were removed such as the one limiting attendance numbers for public gatherings and events.

## New guidelines as of 1 November – stay home if you have symptoms of a respiratory tract infection

Local authorities and municipalities may have additional procedures in place based on the Public Health Agency's guidelines.

### Guidelines for preschool children, the fully vaccinated and those who have tested positive for COVID-19 in the past six months

The following guidelines apply to children who have not started school, fully vaccinated individuals and those who have tested positive for COVID-19 in the past six months:

- They should stay home if they are showing symptoms of a respiratory tract infection. Such symptoms include a sore throat, fever, cough and a general feeling of illness.
- As a rule, this group does not need to get tested for COVID-19.
- People may return to preschool, school, work or leisure activities once they
- have been fever-free for 24 hours and
- feel well, even if they are still displaying signs of a respiratory infection.

In most cases, these people will have to be home for a few days or up to a week.

Individuals and parents and guardians can refer to these guidelines when deciding if they or their child are well enough to return to preschool, school, leisure activities, and work.

### Guidelines for all adults, young people and children of preschool age and above who are not fully vaccinated

The following guidelines apply to everyone of preschool age (around age 6) and above if they are not fully vaccinated:

- They should stay home if they are showing symptoms of a respiratory tract infection. Such symptoms include a sore throat, fever, cough and a general feeling of illness.

- They should take a COVID-19 test as soon as possible.

People who are unvaccinated and who test negative for COVID-19 (are not carriers of the disease) can return to preschool, school, work, and leisure activities if they:

- have been fever-free for 24 hours and
- feel well, even if they are still displaying signs of a respiratory infection.

In most cases, these people will have to be home for a few days or up to a week.

Individuals and parents and guardians can refer to these guidelines when deciding if they or their child are well enough to return to preschool, school, leisure activities, and work.

### People who have tested positive for COVID-19 (carriers of the disease)

Those who have tested positive must stay home and follow the rules in place for people infected with COVID-19. This will not change on 1 November.

COVID-19 information and rules for patients (Smittskyddsläkarföreningen.se)

They may return to preschool, school, work, and leisure activities if they:

- have stayed home for a minimum of 7 days since the onset of their symptoms
- have not had a fever for 48 hours
- feel well, even if they are still displaying signs of a respiratory infection.

## New testing guidelines as of 1 November

Local authorities and municipalities may have additional procedures in place based on the Public Health Agency's guidelines.

### The following people should get tested

- All preschool children (around age 6) and older, young people and adults who are not fully vaccinated and have symptoms that could be a sign of COVID-19.
- Unvaccinated people should continue to get tested following travel to non-Nordic countries, even if they do not have any symptoms. Those who have tested positive for COVID-19 in the last 6 months and children under the age of six are exempt from these requirements.

### The following groups should get tested if they show symptoms of COVID-19, even if they are fully vaccinated

- People who live in sheltered housing or use home help services.
- Older patients living in care homes.
- People who require medical care or treatment for symptoms of COVID-19.
- Medical and healthcare staff.
- Care staff for sheltered accommodation residents or home help workers, and care home staff.
- People who develop symptoms after travel to a non-Nordic country.

Testing conducted as part of contact tracing is more comprehensive and includes both fully vaccinated and unvaccinated people. Read more in the COVID-19 contact tracing guidelines (in Swedish).

### Testing is not recommended for the following groups as of 1 November

These groups should stay home if they develop symptoms, however as of 1 November they will not need to get tested:

- Children, young people and adults who are fully vaccinated, or who have tested positive for COVID-19 in the past six months, and who are not a member of any of the above groups.
- Children who have not started school (under the age of 6).

## Reasons for the changes

COVID-19 prevention measures should not be more restrictive than is justified by the threat to human health. They must also take the best interests of the child into account. Higher vaccination rates reduce the risk of serious illness and disease transmission between and from fully vaccinated people. As a result, the guidelines can be adapted so fully vaccinated people generally do not need to be tested if they develop symptoms.

However, there are still groups that should get tested, such as those who are unvaccinated, at higher risk of becoming seriously ill, or if they spend time close to older people in a risk group.

## The Public Health Agency’s general guidelines changed on 29 September

These general guidelines still apply after 1 November.

### Protect others from the risk of COVID-19 infection

Everyone should stay alert to the symptoms of COVID-19. If you suspect you have COVID-19, you should stay home and avoid contact with other people.

### Limit the spread of COVID-19

Vaccination is the best way to avoid becoming seriously ill and spreading COVID-19.

If you are not fully vaccinated against COVID-19, you should be especially cautious around people in risk groups and those aged 70 or above. You should also take the necessary precautions to avoid catching and spreading COVID-19. Where possible you should:

- keep your distance from other people
- take extra steps to avoid close contact with people in risk groups and those aged 70 and above.

This advice does not apply to people aged under 18 or for those who have been advised against the COVID-19 vaccination on medical grounds.

## Recommendations to those entering Sweden from abroad

The recommendation to get tested upon arrival in Sweden applies up until 31 December. This recommendation applies to those aged 6 or above who are not fully vaccinated or who have not had confirmed COVID-19 in the past 6 months.

### Fact sheet

Have you not been vaccinated yet, or have you only gotten your first dose?

Contact

info@folkhalsomyndigheten.se

About the website

Cookies

Phone: +46 (0)10-205 20 00

Processing of personal data

The Public Health Agency of Sweden is an expert authority with responsibility for public health issues at a national level. The Agency develops and supports activities to promote health, prevent illness and improve preparedness for health threats. Our vision statement: a public health that strengthens the positive development of society.

The Wayback Machine - https://web.archive.org/web/20211130005037/https://www.folkhalsomyndi…


# COVID-19 infection control measures as from 22 November and 1 December

**The recommendations for testing will be extended as from 22 November. On 1 December more infection control measures will be introduced, such as a possibility of using vaccination certificates at public gatherings and events, and recommendations to operators to make it easier for visitors to keep a distance.**

## This applies as from 22 November

### Both those who are vaccinated and those who are not vaccinated should get tested when they experience symptoms

Also those who are vaccinated are recommended to get tested when they experience symptoms. The following recommendations apply:

- Pay attention to new symptoms such as a sore throat, runny nose, fever, cough, or feeling generally unwell.
- Stay home if you develop any of these symptoms, or other symptoms of a respiratory infection.
- Get tested for COVID-19 if you develop symptoms. This applies both to those who are vaccinated and to those who are not vaccinated. Children who have not yet started preschool class and those who have had a

confirmed COVID-19 infection in the past six months do not need to get tested. At 1177.se you can read about testing procedures in the region where you live.

- By staying home when you develop symptoms you reduce the spread of COVID-19 and other infections such as influenza and the respiratory syncytial virus (RSV).
- Stay home from work, school, preschool and other activities when you have symptoms of a respiratory infection even if you have received a negative test result for COVID-19. It will reduce the spread of other infections.
- Call telephone number 1177 if you need help assessing your symptoms or advice on where to seek medical care. This is particularly important for people in a risk group who may need care for diseases other than COVID-19, e.g. influenza.

## This applies as from 24 November

The guidelines for contact tracing, issued by the Public Health Agency, have been updated. Members of a household with a person diagnosed with COVID-19 must stay home for seven days and get tested for COVID-19.

### This applies:

When a person tests positive for COVID-19, those who live in the same household will get rules of conduct from the health care services about staying home for seven days from the day on which the person who tested positive took the test. This applies both to those who are vaccinated and to those who are not vaccinated, regardless of whether you have symptoms or not and regardless of age.

Everyone is recommended to get tested. Those who experience symptoms are recommended to get tested immediately when symptoms appear. Those who do not have any symptoms should get tested five days after the person in the household who tested positive took the test. Children who have not yet started preschool class do not need to get tested, but should stay home.

Anyone who does not experience symptoms and has received a negative test result can return to work, school or other activities seven days after the day on which the person in the household who tested positive took the test. It is still important to pay attention to new symptoms.

People who have had a confirmed COVID-19 infection in the past six months do not need to get tested or stay home in connection with contact tracing. However, they need to stay home if they experience new symptoms of respiratory infection.

The recommendation for vaccinated people and for those who have no symptoms to stay home and get tested if anyone in the household test positive for COVID-19 will be introduced in the regions gradually.

## This applies as from 1 December

As from 1 December new regulations and general guidelines against the spread of infection at public gatherings and events apply. They cover public gatherings and public events, and include environments where many people gather. From 1 December it will be possible for organisers of indoor public gatherings and events with more than 100 participants to use vaccination certificates.

## Public gatherings and public events with vaccination certificates

Indoor public gatherings and public events with more than 100 participants can either use vaccination certificates or apply more extensive infection control measures. If they use vaccination certificates, they still have to comply with the basic infection control measures. These include putting up information on how to avoid the spread of infection, providing the possibility of hand washing and informing staff. They also need to have procedures for handling vaccination certificates. People under the age of 18 do not need to present a vaccination certificate.

By requiring vaccination certificates from participants, an organiser can continue to operate without limiting the number of participants.

The possibility to use vaccination certificates with be introduced on 1 December.

# Public gatherings and public events without vaccination certificates

Indoor public gatherings and public events with more than 100 participants that do not use vaccination certificates need to take more infection control measures.

These include following the basic infection control measures, such as informing visitors about how to avoid the spread of infection and providing the possibility of hand washing. They also need to offer visitors an assigned seat, have a maximum of eight people in each party, and at least one metre distance between groups.

They are also covered by the general guidelines to avoid crowding. These include spreading participants' arrival time, having specific entrances and exits, marking distances and have other alternatives to physical queues.

# Indoor fairs are covered by the regulation and general guidelines

Indoor fairs are covered by the regulation and general guidelines against the spread of infection. These include providing information on how to avoid the spread of infection, offering a possibility of washing hands and designing passages between for example stands to avoid crowding. Foreign visitors also need to be informed about the Public Health Agency's recommendations.

# Activities covered by the general guidelines against the spread of infection

Leisure and cultural activities, trading venues and markets are also covered by the general guidelines but not by the possibility of using vaccination certificates.

They have to inform their visitors about how to avoid the spread of infection and to provide the possibility of hand washing. They are also recommended to take measures to reduce the risk of crowding. These include alternatives to queues, spreading visitors' arrival time, guide visitors to apply an even throughput of people at markets, or marking an appropriate distance on the floor.

# Vaccination certificate requirements

Vaccination is a prerequisite for a vaccination certificate and cannot be replaced by a certificate of recovery or a negative test result.

In Sweden “vaccinated” means having received at least two doses of a COVID-19 vaccine. At least two weeks must have passed since the second dose. People who are not vaccinated or have only received one dose are considered unvaccinated and cannot get a vaccination certificate.

You can download your vaccination certificate at E-hälsomyndigheten

## Reasons for the changes

COVID-19 prevention measures should not be more restrictive than is justified by the threat to human health. They must also take the best interests of the child into account. The Public Health Agency is therefore continuously adapting the measures against COVID-19 to the current situation. In mid-November, a rise has been seen in European countries despite high vaccination coverage, and there has been an increasing spread of infection in some Swedish regions. The Public Health Agency therefore considers that further actions are needed.

## The Public Health Agency’s general guidelines as from 29 September

### Protect others from the risk of COVID-19 infection

Everyone should stay alert to the symptoms of COVID-19. If you suspect you have COVID-19, you should stay home and avoid contact with other people.

### Limit the spread of COVID-19

Vaccination is the best way to avoid becoming seriously ill and spreading COVID-19.

If you are not fully vaccinated against COVID-19, you should be especially cautious around people in risk groups and those aged 70 or above. You should also take the necessary precautions to avoid catching and spreading COVID-19. Where possible you should:

- keep your distance from other people

- take extra steps to avoid close contact with people in risk groups and those aged 70 and above.

This advice does not apply to people aged under 18 or for those who have been advised against the COVID-19 vaccination on medical grounds.

## Recommendations to those entering Sweden from abroad

The recommendation to get tested upon arrival in Sweden applies up until 31 December. This recommendation applies to those aged 6 or above who are not fully vaccinated or who have not had confirmed COVID-19 in the past 6 months.

Recommendation for all travellers to Sweden to get tested if you develop symptoms of COVID-19

### Fact sheet

Have you not been vaccinated yet, or have you only gotten your first dose?

### Contact

info@folkhalsomyndigheten.se

Phone: +46 (0)10-205 20 00

### About the website

Cookies

Processing of personal data

The Public Health Agency of Sweden is an expert authority with responsibility for public health issues at a national level. The Agency develops and supports

activities to promote health, prevent illness and improve preparedness for health threats. Our vision statement: a public health that strengthens the positive development of society.

The Wayback Machine - https://web.archive.org/web/20211205233825/https://www.regionorebrola...

Region Örebro län

Välkommen till Region Örebro län! › Aktuellt och nyheter

# Lägesbild corona vecka 42: Rekommendationerna kring testning uppdateras

Publicerad: 2021-10-19 14:52

Smittspridningen av covid-19 i Örebro län har legat på ungefär samma nivå de senaste tre veckorna. Förra veckan hade länet cirka 170 positiva fall. Det kan jämföras med cirka 180 fall veckan innan.

- Åldersfördelning är den samma som tidigare. Drygt hälften av fallen finns i åldrarna 6-19 år, flest smittade finns bland dem som är 6-15 år. Det kan förklaras med att flest ovaccinerade finns i de här åldrarna, säger Gunlög Rasmussen, smittskyddsläkare.

Större delen av befolkningen i Sverige är nu fullvaccinerad. Det innebär en minskad risk för allvarlig sjukdom i covid-19 och även minskad belastning på vården. Med anledning av detta kommer de nationella rekommendationerna kring testning uppdateras från och med den 1 november. Fokus kommer därefter att ligga på miljöer och grupper där smittspridning fortsatt kan få stora konsekvenser.

- Regionen följer de nationella riktlinjerna. Det innebär att fullvaccinerade länsbor från och med den 1 november inte längre behöver testas vid luftvägssymtom och feber. De som haft covid-19 senaste halvåret är också generellt undantagna testningen, säger Gunlög Rasmussen.

### Ovaccinerade rekommenderas fortsatt testning vid symtom

Ovaccinerade personer från förskoleklass och uppåt rekommenderas däremot testning vid symtom precis som tidigare. För personal inom vård och omsorg är rekommendationerna också oförändrade. De behöver ta prov vid symtom oavsett om de är vaccinerade eller ej.

Provtagning i samband med smittspårning, efter att en person blivit utsatt för smitta, sker som tidigare utifrån särskilda rekommendationer. Rekommendationen att ovaccinerade ska testa sig efter utlandsresa utanför Norden är också kvar.

- Det är också viktigt att komma ihåg att alla ska fortsätta stanna hemma vid symtom på luftvägsinfektion, oavsett vaccination, inte bara för att minska risken för covid-19, utan också för att minska risken för att andra luftvägsinfektioner, som RS-virus och framöver influensa, sprids. Fullvaccinerade personer och de som haft covid-19, liksom barn i förskoleåldern, kan sedan återvända till arbete, skola och förskola om de har varit feberfria i minst ett dygn och känner sig friska, säger Gunlög Rasmussen.

### Vaccinationstäckningen är fortsatt hög i Örebro län

218 328 personer i Örebro län har fått den första dosen vaccin mot covid-19. Det är drygt 83,43 procent av befolkningen i regionen. 210 293 personer har fått dos 2, vilket är drygt 80,39 procent. 5 022 personer har fått en tredje dos. Det motsvarar 1,99 procent av länets befolkning.

- Vaccinationstäckningen är hög i länet. Rekommendationen är att du bokar tid för vaccinering för då är du garanterad att det finns vaccin, säger Stellan Johansson, vaccinationskoordinator.

Majoriteten av länets kommuner kommer att erbjuda vaccin via skolan till de som fyllt 12-15 år. Detta kommer att påbörjas under de närmaste veckorna.

- Alla vårdnadshavare, till elever på de skolor som erbjuder vaccinationer, kommer att få ett brev från skolan med erbjudande om vaccinering. I brevet finns samtyckesblankett, hälsodeklaration och övrig information. Svarskuvert med ifylld samtyckesblankett och hälsodeklaration ska eleverna ha med sig tillbaka till skolan, säger Stellan Johansson.

Vårdnadshavare som vill vaccinera sitt barn tidigare kan antingen boka tid eller gå på drop in på en vaccinationsmottagning.

### Personer födda 1991 eller senare ska avvakta besked

De personer som är födda 1991 eller senare, och som fått en dos av Modernas vaccin, har fått ett SMS från Region Örebro län om att tills vidare inte ta en andra dos, utan invänta ytterligare besked.

- Vi väntar på mer information från Folkhälsomyndigheten om hur vaccinationen för dessa personer ska fortsätta och återkommer så fort vi vet mer, säger Stellan Johansson.

---

### Rekommendationer som fortfarande gäller:

Vaccinera dig – det är bästa sättet att undvika allvarlig sjukdom och spridning av covid-19.

Var uppmärksam på symtom på covid-19. Vid symtom på covid-19 ska du stanna hemma och testa dig. Gäller även fullvaccinerade fram till och med den 31 oktober. Barn under 6 år behöver inte testa sig.

Vuxna som kan vaccinera sig, men ännu inte gjort det, bör fortsätta hålla avstånd och ta särskild hänsyn till personer i riskgrupp och de som är över 70 år.

---

Senast uppdaterad: den 19 oktober 2021

The Wayback Machine - https://web.archive.org/web/20211206232406/https://www.regiondalarna.se/press/nyheter-och-pressmeddela…



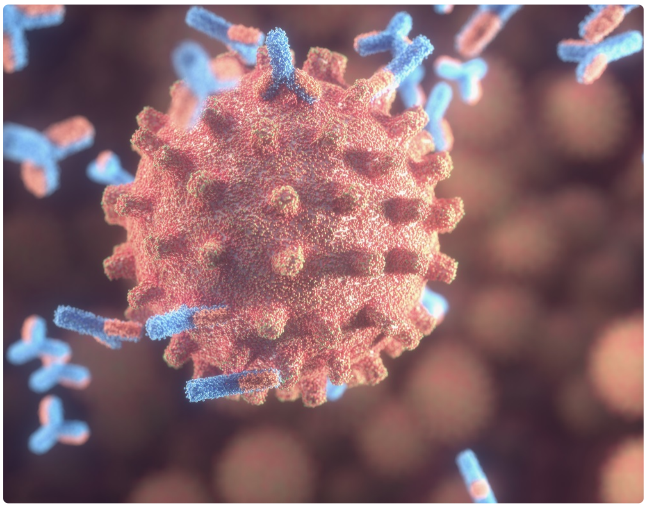

# Nu behöver inte fullvaccinerade med covid-19-symtom prov-ta-sig

**Pressmeddelande** – 1 november 2021

**– Det här är en sjukdom vi måste leva med. Nu är många fullvaccinerade och omfattande testning bedöms inte längre vara motiverat – men vi behöver fortfarande se till att smittan inte kommer in i känsliga miljöer för att skydda sårbara grupper, säger Helena Ernlund, biträdande smittskyddsläkare.**

Fullvaccinerade barn, unga och vuxna behöver från och med den 1 november generellt inte testas för covid-19 vid nytillkomna luftvägssymtom eller feber. Detsamma gäller den som det senaste halvåret haft en bekräftad covid-19-infektion.

– Det är alltid viktigt att stanna hemma från jobb, skola och förskola vid nytillkomna förkylningssymtom eller feber. Bara på så sätt minskar vi risken allmänt för smittspridning av olika luftvägsinfektioner som till exempel RS-virus och influensa, säger Helena Ernlund.

Folkhälsomyndighetens nya rekommendation sker mot bakgrund av att stora delar i befolkningen över 16 år nu är fullvaccinerade. I Dalarna är cirka 83 procent över 16 år fullvaccinerade och hela 87 procent har fått dos ett.

Den höga vaccinationstäckningen i landet minskar risken för omfattande smittspridning och gör att färre människor drabbas av allvarliga konsekvenser till följd av covid-19-infektioner.

– Det viktigaste nu är att vi testar i miljöer och grupper där det finns risk för allvarliga konsekvenser av covid-19, säger Helena Ernlund.

Testning rekommenderas fortsättningsvis vid symtom:

- För alla ovaccinerade personer från förskoleklass och uppåt som inte nyligen genomgått covid-19.
- För vård- och omsorgspersonal
- För äldreomsorgstagare, äldre inom LSS-verksamhet samt vid behov av vård och behandling för misstänkt covid-19.

- Personer som får symptom efter resa i ett land utanför Norden.

Den som är fullvaccinerad och vill prov-ta-sig vid symtom kan även fortsättningsvis göra det.

Testning rekommenderas fortsättningsvis även utan symtom:

- För ovaccinerade personer som inte genomgått covid-19 under det senaste halvåret efter vistelse i land utanför Norden.
- Om du ingår i en smittspårning.

Som tidigare gäller att små barn i förskoleåldern inte testas.

**Hur länge ska man vara hemma efter att haft en luftvägsinfektion om man är fullvaccinerad?**
– Man ska vara hemma tills man varit feberfri i minst ett dygn och känner sig frisk. Det är okej att gå tillbaka till skola eller arbete om man har vissa luftvägssymtom kvar, så länge man har förbättrats.
– Detsamma gäller för den som är ovaccinerad och har genomgått ett test som var negativt, säger Helena Ernlund.

## Kontaktpersoner

**Presstjänst**
Presstjänst
010-249 90 20
kommunikation.presstjanst@regiondalarna.se

Läs pressmeddelandet på mynewsdesk.com

Tillbaka

Dela:

The Wayback Machine - https://web.archive.org/web/20211102174548/https://www.regionsto...

**Cookies**

På Region Stockholms webbplats använder vi cookies för att ge dig en bättre upplevelse av webbplatsen. Genom att surfa vidare godkänner du att vi använder cookies.

Läs mer om cookies (https://web.archive.org/web/20211102174548/https://www.regionstockholm.se/om-webbplatsen/cookies/)

Jag godkänner ×

Region Stockholm

# 2 november: lägesrapport om covid-19

**Nyhet - Hälsa och vård**
Publicerad: 2021-11-02

1 618 nya fall av covid-19 har konstaterats under perioden 26 oktober till och med 1 november. Förra tisdagen rapporterades 1 355 konstaterade fall. Totalt vårdas 74 personer för covid-19 vid något av regionens sjukhus. Det innebär att det är 7 färre patienter med covid-19 i behov av sjukhusvård jämfört med tisdag i förra veckan.

- Liksom förra veckan ser vi en viss ökning av antalet konstaterat smittade med covid-19. Det är fortfarande på låga nivåer men likväl oroväckande att fler smittas. Nu hoppas jag att de som smittas får ett lindrigt sjukdomsförlopp och slippa behöva vårdas vid något av våra sjukhus, säger Region Stockholms smittskyddsläkare Maria Rotzén Östlund.

- Den fortsatt största utmaningen är att få fler att vaccinera sig i områden och grupper med lägre vaccinationstäckning, säger Maria Rotzén Östlund.

## Antal konstaterat smittade

1 618 nya fall av covid-19, varav 13 var provtagna på ett särskilt boende för äldre, har konstaterats genom provtagning under perioden 26 oktober och 1 november.

Sedan pandemins början har 280 692 fall av covid-19 konstaterats i länet.

## Samlad veckostatistik

Under hela vecka 43 konstaterades 1 583 nya fall av covid-19. Veckan innan, vecka 42, konstaterades 1 484 nya fall.

Original text

Från och med vecka 42 har SmiNet öppet även på helger. Detta gav som resultat att i den data som presenteras för vecka 42 så ingår måndag till söndag vecka 42 - men även lördagen och söndagens data för vecka 41. Vecka 42 har data för 9 dagar istället för sju dagar.

Contribute a better translation

## Provtagning

Under vecka 43 har det analyserats 22 987 tester för pågående covid-19. Av dessa var 1 757 provsvar eller 7,7 procent positiva. Andelen positiva provsvar är högre jämfört med föregående vecka då andelen positiva provsvar var 6,3 procent.

Från och med den här veckan har Folkhälsomyndigheten förändrat provtagningsindikationerna. Det kommunicerades tidigare och kan till del vara förklaring till ett lägre antal provtagningar.

Efterfrågan på bokningsbara tider för PCR-provtagning minskade något jämfört med föregående vecka. Kapaciteten för PCR-provtagning är fortsatt god, kapaciteten är större än efterfrågan. Patienter, medarbetare och boende på äldreboenden och personal inom vård och omsorg har egna provtagningsprocesser och berörs inte om köer i den storskaliga provtagningen skulle uppstå.

## Antal vårdade

Totalt vårdas just nu 74 patienter med konstaterad covid-19 på akutsjukhus eller geriatrisk vård.

Av dessa vårdas:

5 i intensivvård
52 på akutsjukhus
17 geriatrisk vård utanför akutsjukhus

Som mest vårdades 1 100 personer i någon form av sjukhusvård (april 2020).

Fram till 1 november har 18 201 patienter skrivits ut till sina hem, vissa av dem med fortsatt vård i hemmet eller till särskilt boende för äldre (SÄBO).

## Antal avlidna

Sedan pandemins början har 4 489 personer med bekräftad covid-19 avlidit. Det är 17 fler än vad som rapporterade förra tisdagen. Av dessa är det 1 651 personer som är provtagna på ett särskilt boende för äldre. Det är 4 fler än förra tisdagen.

Det finns en eftersläpning i inrapporteringen av avlidna.

## Vaccination mot covid-19

Vaccinationsläget 2 november

Nu redovisas registrerade vaccinationer utifrån ålder i relation till den befolkningsstatistik från SCB som Folkhälsomyndigheten redovisar.

Antal registrerade vaccinationer dos 1: 1 618 358
Antal registrerade vaccinationer dos 2: 1 471 961
Antal registrerade vaccinationer dos 3: 45 125
Totalt antal registrerade vaccinationer: 3 135 444

Andel vaccinerade av befolkningen i Region Stockholm som är över 18 år:

Andel dos 1: 78,7 procent
Andel dos 2: 74,3 procent

Andel vaccinerade av befolkningen i Region Stockholm som är över 12 år:
Andel dos 1: 75,4 procent
Andel dos 2: 69,4 procent

Andel givna dos 1 i region Stockholm av befolkningen som är över 18 år, baserat på befolkningsunderlag 31/12–2020:
Andel dos 1: 81,7 procent
Andel dos 2: 77,0 procent

Andel vaccinerade vid SÄBO:
Dos 1: 94,5 procent
Dos 2: 90,1 procent
Dos 3: 62,2 procent

Andel vaccinerade som är över 80 år:
Dos 1: 93,3 procent
Dos 2: 90,7 procent
Dos 3: 29,4 procent

Andel vaccinerade som är 75–79 år:
Dos 1: 91,9 procent
Dos 2: 89,4 procent

Andel vaccinerade som är 70–74 år:
Dos 1: 88,0 procent
Dos 2: 85,8 procent

Andel vaccinerade som är 65–69 år:
Dos 1: 85,9 procent
Dos 2: 83,5 procent

Totalt andel vaccinerade som är över 65 år
Dos 1: 89,7 procent
Dos 2: 87,2 procent

Andel vaccinerade som är 60–64 år:
Dos 1: 86,9 procent
Dos 2: 84,7 procent

Andel vaccinerade som är 55–59 år:
Dos 1: 86,7 procent
Dos 2: 84,1 procent

Andel vaccinerade som är 50–54 år:
Dos 1: 85,4 procent
Dos 2: 82,2 procent

Andel vaccinerade som är 45–49 år:
Dos 1: 83,1 procent
Dos 2: 78,8 procent

Andel vaccinerade som är 40–44 år:
Dos 1: 78,3 procent
Dos 2: 73,2 procent

Andel vaccinerade som är 35–39 år:
Dos 1: 73,7 procent
Dos 2: 68,2 procent

Andel vaccinerade som är 30–34 år:
Dos 1: 68,6 procent
Dos 2: 63,6 procent

Andel vaccinerade som är 18–29 år:
Dos 1: 63,6 procent
Dos 2: 56,0 procent

Andel vaccinerade som är 16–17 år:
Dos 1: 64,9 procent
Dos 2: 45,7 procent

Andel vaccinerade som är 12–15 år:
Dos 1: 26,6 procent
Dos 2: 1,7 procent

Vårdgivarna registrerar antalet vaccinationer i vårt digitala verktyg för vaccinationer. Det är upp till sju dagars eftersläpning i inrapporteringen. Därför är det fler som faktiskt är vaccinerade än vad som syns i vår rapportering.

Andel över 65, 40 och 18 år som påbörjat vaccinationen samt andel fullvaccinerade över 18 år fördelat per kommun/stadsdelsområde

| Kommun/Stadsdel | Andel +65 | Andel +40 | Andel +18 | Andel fullvaccinerade |
|---|---|---|---|---|

| | | | | |
|---|---|---|---|---|
| **Bromma** | 90,6% | 89,1% | 83,5% | 79,1% |
| **Enskede-Årsta-Vantör** | 87,4% | 83,6% | 77,8% | 73,0% |
| **Farsta** | 89,3% | 84,8% | 79,4% | 74,4% |
| **Hägersten-Älvsjö** | 88,8% | 88,0% | 82,8% | 78,8% |
| **Hässelby-Vällingby** | 89,8% | 83,7% | 75,3% | 70,1% |
| **Kungsholmen** | 87,6% | 87,3% | 81,8% | 79,3% |
| **Norrmalm** | 86,7% | 85,1% | 79,3% | 77,1% |
| **Rinkeby Kista** | 82,2% | 72,1% | 60,3% | 54,3% |
| **Skarpnäck** | 87,2% | 85,7% | 81,2% | 77,1% |
| **Skärholmen** | 83,4% | 75,2% | 65,2% | 59,2% |
| **Spånga Tensta** | 86,6% | 79,7% | 68,3% | 62,7% |
| **Södermalm** | 87,9% | 88,0% | 83,6% | 80,2% |
| **Östermalm** | 86,4% | 86,0% | 77,5% | 75,1% |
| **Botkyrka** | 86,0% | 77,7% | 67,6% | 62,0% |
| **Danderyd** | 91,5% | 91,0% | 85,9% | 81,7% |
| **Ekerö** | 93,2% | 89,6% | 85,2% | 80,7% |
| **Haninge** | 89,9% | 83,4% | 75,0% | 69,3% |
| **Huddinge** | 89,4% | 84,5% | 76,4% | 71,3% |

| | | | | |
|---|---|---|---|---|
| **Järfälla** | 91,5% | 86,1% | 77,1% | 72,2% |
| **Lidingö** | 90,9% | 89,5% | 84,6% | 80,2% |
| **Nacka** | 91,1% | 89,2% | 84,0% | 79,4% |
| **Norrtälje** | 95,0% | 91,3% | 86,2% | 82,0% |
| **Nykvarn** | 92,1% | 89,0% | 82,7% | 78,9% |
| **Nynäshamn** | 92,5% | 87,4% | 80,7% | 74,9% |
| **Salem** | 91,1% | 87,6% | 80,1% | 75,0% |
| **Sigtuna** | 91,0% | 83,3% | 73,7% | 68,7% |
| **Sollentuna** | 91,6% | 88,4% | 82,2% | 78,3% |
| **Solna** | 88,4% | 85,5% | 77,2% | 73,5% |
| **Sundbyberg** | 88,2% | 83,7% | 75,7% | 71,3% |
| **Södertälje** | 87,6% | 78,3% | 67,7% | 62,9% |
| **Tyresö** | 92,6% | 89,1% | 83,2% | 78,1% |
| **Täby** | 92,6% | 91,6% | 87,2% | 83,6% |
| **Upplands Bro** | 91,0% | 84,6% | 75,7% | 69,6% |
| **Upplands Väsby** | 91,2% | 85,6% | 78,0% | 73,3% |
| **Vallentuna** | 93,8% | 90,4% | 85,5% | 81,4% |
| **Vaxholm** | 92,9% | 91,5% | 88,3% | 84,2% |

| | | | | |
|---|---|---|---|---|
| **Värmdö** | 93,2% | 90,2% | 85,4% | 80,3% |
| **Österåker** | 93,6% | 90,1% | 85,6% | 81,0% |
| ***Saknas*** | *42,0%* | *60,2%* | *50,2%* | *45,0%* |
| **Totalt** | **89,7%** | **85,8%** | **78,7%** | **74,3%** |

## Hur vi rapporterar

Region Stockholm rapporterar om covid-läget på tisdagar och fredagar

Uppgifterna om antalet sjukhusvårdade är en ögonblicksbild från samma dag som rapporten publiceras.

På grund av kvalitetssäkring av uppgifterna om avlidna finns det en eftersläpning i inrapporteringen.

Ett viktigt mål med rapporten är att publicera uppgifter så snabbt som möjligt, både efterregistreringar och korrigeringar kan komma att ske.

## Vill du prenumerera på liknande nyheter?

Din e-postadress

### Välj kategori

Hälsa och vård

Aktivera prenumerationen

Se fler kategorier samt läs om hur vi hanterar dina personuppgifter på prenumerationssidan ›

Hitta rätt i vården (https://web.archive.org/web/20211102174548/http://www.1177.se/Stockholm/)

Planera din resa (https://web.archive.org/web/20211102174548/https://sl.se/)

Färdtjänst och sjukresor (https://web.archive.org/web/20211102174548/http://www.fardtjansten.sll.se/)

Vår vision är en attraktiv, hållbar och växande stockholmsregion med frihet för invånarna att själva forma sina liv och fatta avgörande beslut.

## Om webbplatsen

- Om webbplatsen (/web/20211102174548/https://www.regionstockholm.se/om-webbplatsen/)
- Prenumerera på nyheter (/web/20211102174548/https://www.regionstockholm.se/Prenumerationssida/)
- Om cookies (/web/20211102174548/https://www.regionstockholm.se/om-webbplatsen/cookies/)
- Tillgänglighetsredogörelse (/web/20211102174548/https://www.regionstockholm.se/om-webbplatsen/tillganglighetsredogorelse/)

## Om Region Stockholm

**Telefon:** 08-737 25 00

**E-post:** kontakt@regionstockholm.se

Kontakta Region Stockholm (/web/20211102174548/https://www.regionstockholm.se/kontakt/kontakta-region-stockholm/)

Anslagstavla (/web/20211102174548/https://www.regionstockholm.se/politik/Anslagstavla/)

## Följ oss i sociala medier

- Facebook
- LinkedIn
- Instagram
- Youtube
- Twitter

Region Stockholm

# 23 november: Lägesrapport om covid-19

**Nyhet - Hälsa och vård**
Publicerad: 2021-11-23

Totalt vårdas 75 personer för covid-19 vid något av regionens sjukhus. Det innebär att det är en patient mer med covid-19 i behov av sjukhusvård jämfört med tisdag i förra veckan. Antalet smittade är 1 930 under perioden 16 november och 22 november vilket är fler än föregående tisdag då vi rapporterade 1 557 smittade.

– Vi ser en ökad smittspridning i regionen, det oroar. Särskilt med tanke på att smittan ökar dramatiskt i många grannländer, säger Johan Bratt, chefläkare vid Region Stockholm.

– Antalet patienter med covid-19 som är inlagda vid våra sjukhus har under flera veckor varit mellan 70 och 90. Antalet covid-vårdade vid sjukhusen kan dock ändra sig eftersom det dröjer ett par veckor mellan det att en person smittad med covid-19 blir så sjuk att hon eller han måste läggas in, säger Johan Bratt.

## Antal konstaterat smittade

1 930 nya fall av covid-19, varav 2 var provtagna på ett särskilt boende för äldre, har konstaterats genom provtagning under perioden 15 november och 21 november.

Sedan pandemins början har 285 413 fall av covid-19 konstaterats i länet.

### Samlad veckostatistik

Under hela vecka 46 konstaterades 1 791 nya fall av covid-19. Veckan innan, vecka 45, konstaterades 1 516 nya fall.

## Provtagning

Under vecka 46 har det analyserats 23 981 tester för pågående covid-19. Av dessa var 1 973 provsvar eller 8,4 procent positiva. Andelen positiva provsvar är något lägre jämfört med föregående vecka då andelen positiva provsvar var 8,9 procent. Region Stockholms mobila provtagningsbussar för assisterad provtagning finns i områden med lägre vaccinationstäckning. Under vecka 46 var 17,7 % av de genomförda testerna i bussarna positiva.

[illegible]en 22 november har Folkhälsomyndigheten beslutat om nya rekommendationer för testning för covid-19.

De nya rekommendationerna – att testa sig vid symtom och att stanna hemma - gäller oavsett om du är vaccinerad eller ovaccinerad. De som undantas är personer som de senaste 6 månaderna har haft covid-19 och tillfrisknat. Eftersom rekommendationerna förändrats både 1 november och 22 november är statistiken inte är direkt jämförbar med tidigare veckor.

### Antal vårdade

Totalt vårdas just nu 75 patienter med konstaterad covid-19 på akutsjukhus eller geriatrisk vård.

Av dessa vårdas:

- 4 i intensivvård
- 55 på akutsjukhus
- 16 geriatrisk vård utanför akutsjukhus

Som mest vårdades 1 100 personer i någon form av sjukhusvård (april 2020).

Fram till 1 november har 18 381 patienter skrivits ut till sina hem, vissa av dem med fortsatt vård i hemmet eller till särskilt boende för äldre (SÄBO).

### Antal avlidna

Sedan pandemins början har 4 519 personer med bekräftad covid-19 avlidit. Det är 8 fler än vad som rapporterade förra tisdagen. Av dessa är det 1 656 personer som är provtagna på ett särskilt boende för äldre. Det är en mer än förra tisdagen.

Det finns en eftersläpning i inrapporteringen av avlidna.

## Vaccination mot covid-19

### Vaccinationsläget 23 november

Under veckan har 50 000 personer som är 65+ fått påfyllnadsdos. Det innebär att nästan var tredje person (32,6 %) i den gruppen nu fått ett förstärkt skydd framförallt mot att bli allvarligt sjuk med covid-19, säger Magnus Thyberg, vaccinationssamordnare i Region Stockholm.

Vi arbetar nu med att förstärka kapaciteten ytterligare. Det är många som erbjuds – och vill – vaccinera sig just nu. Min uppmaning är att gå in Alltid Öppet-appen och se om det finns tider som passar.

Alla som bor på SÄBO och som har tackat ja till en påfyllnadsdos har nu fått den. I sifferdelen nedan anges en lägre siffra för andelen vaccinerade med en tredje dos på SÄBO. Det beror på eftersläpning i rapporteringen.

Nu redovisas registrerade vaccinationer utifrån ålder i relation till den befolkningsstatistik från SCB som Folkhälsomyndigheten redovisar.

Antal registrerade vaccinationer dos 1: 1 649 015
Antal registrerade vaccinationer dos 2: 1 505 794
Antal registrerade vaccinationer dos 3: 146 948
Totalt antal registrerade vaccinationer: 3 301 757

Andel vaccinerade av befolkningen i Region Stockholm som är över 18 år:

Andel dos 1: 79,2 procent
Andel dos 2: 75,4 procent

Andel vaccinerade av befolkningen i Region Stockholm som är över 12 år:
Andel dos 1: 76,7 procent
Andel dos 2: 70,9 procent

Andel givna dos 1 i region Stockholm av befolkningen som är över 18 år, baserat på befolkningsunderlag 31/12–2020:
Andel dos 1: 82,6 procent
Andel dos 2: 78,3 procent

Andel vaccinerade vid SÄBO:
Dos 1: 94,8 procent
Dos 2: 90,8 procent
Dos 3: 72,6 procent

Andel vaccinerade som är över 80 år:
Dos 1: 93,5 procent
Dos 2: 91,4 procent
Dos 3: 57,2 procent

Andel vaccinerade som är 75–79 år:
Dos 1: 92,0 procent
Dos 2: 89,8 procent

Andel vaccinerade som är 70–74 år:
Dos 1: 88,2 procent
Dos 2: 86,2 procent

Andel vaccinerade som är 65–69 år:
Dos 1: 86,1 procent
Dos 2: 83,9 procent

Totalt andel vaccinerade som är över 65 år
Dos 1: 89,9 procent
Dos 2: 87,7 procent
Dos 3: 32,6 procent

Andel vaccinerade som är 60–64 år:
Dos 1: 87,2 procent
Dos 2: 85,1 procent

Andel vaccinerade som är 55–59 år:
Dos 1: 87,0 procent
Dos 2: 84,6 procent

Andel vaccinerade som är 50–54 år:
Dos 1: 85,7 procent
Dos 2: 82,8 procent

Andel vaccinerade som är 45–49 år:
Dos 1: 83,6 procent
Dos 2: 79,5 procent

Andel vaccinerade som är 40–44 år:
Dos 1: 78,8 procent
Dos 2: 74,2 procent

Andel vaccinerade som är 35–39 år:
Dos 1: 74,5 procent
Dos 2: 69,5 procent

Andel vaccinerade som är 30–34 år:
Dos 1: 69,4 procent
Dos 2: 65,3 procent

Andel vaccinerade som är 18–29 år:
Dos 1: 64,6 procent
Dos 2: 58,4 procent

Andel vaccinerade som är 16–17 år:
Dos 1: 66,6 procent
Dos 2: 50,7 procent

Andel vaccinerade som är 12–15 år:
Dos 1: 41,0 procent
Dos 2: 9,0 procent

Vårdgivarna registrerar antalet vaccinationer i vårt digitala verktyg för vaccinationer. Det är upp till sju dagars eftersläpning i inrapporteringen. Därför är det fler som faktiskt är vaccinerade än vad som syns i vår rapportering.

## Andel över 65, 40 och 18 år som påbörjat vaccinationen samt andel fullvaccinerade över 18 år fördelat per kommun/stadsdelsområde

| Kommun/Stadsdel | Andel +65 | Andel +40 | Andel +18 | Andel fullvaccinerade +18 |
|---|---|---|---|---|
| Bromma | 90,9% | 89,3% | 83,9% | 80,1% |
| Enskede-Årsta-Vantör | 87,6% | 84,0% | 78,4% | 74,2% |
| Farsta | 89,5% | 85,1% | 79,9% | 75,6% |
| Hägersten-Älvsjö | 88,9% | 88,3% | 83,2% | 79,8% |
| Hässelby-Vällingby | 90,2% | 84,1% | 76,1% | 71,4% |
| Kungsholmen | 87,8% | 87,5% | 82,1% | 80,3% |
| Norrmalm | 86,8% | 85,3% | 79,7% | 78,1% |
| Rinkeby-Kista | 82,4% | 72,8% | 61,3% | 55,9% |
| Skarpnäck | 87,3% | 85,9% | 81,6% | 78,1% |
| Skärholmen | 83,8% | 75,8% | 66,2% | 60,8% |
| Spånga-Tensta | 86,9% | 80,3% | 69,2% | 64,0% |
| Södermalm | 88,1% | 88,2% | 83,9% | 81,0% |
| Östermalm | 86,5% | 86,2% | 77,9% | 76,2% |
| Botkyrka | 86,3% | 78,3% | 68,5% | 63,4% |
| Danderyd | 91,7% | 91,2% | 86,2% | 82,6% |

| | | | | |
|---|---|---|---|---|
| Ekerö | 93,3% | 89,8% | 85,6% | 81,6% |
| Haninge | 90,1% | 83,8% | 75,6% | 70,6% |
| Huddinge | 89,6% | 84,9% | 77,0% | 72,5% |
| Järfälla | 91,7% | 86,5% | 77,7% | 73,4% |
| Lidingö | 91,0% | 89,7% | 84,9% | 81,1% |
| Nacka | 91,3% | 89,5% | 84,5% | 80,5% |
| Norrtälje | 95,1% | 91,4% | 86,5% | 82,8% |
| Nykvarn | 92,2% | 89,2% | 83,0% | 79,9% |
| Nynäshamn | 92,7% | 87,7% | 81,0% | 75,8% |
| Salem | 91,2% | 87,9% | 80,7% | 76,1% |
| Sigtuna | 91,2% | 83,6% | 74,3% | 69,9% |
| Sollentuna | 91,7% | 88,7% | 82,6% | 79,2% |
| Solna | 88,5% | 85,8% | 77,7% | 74,7% |
| Sundbyberg | 88,3% | 84,1% | 76,3% | 72,6% |
| Södertälje | 87,8% | 78,8% | 68,5% | 64,3% |
| Tyresö | 92,7% | 89,3% | 83,7% | 79,3% |
| Täby | 92,8% | 91,8% | 87,5% | 84,4% |
| Upplands Bro | 91,1% | 85,0% | 76,5% | 71,0% |

| | | | | |
|---|---|---|---|---|
| Upplands Väsby | 91,4% | 85,9% | 78,6% | 74,4% |
| Vallentuna | 94,0% | 90,6% | 85,9% | 82,2% |
| Vaxholm | 93,0% | 91,7% | 88,6% | 85,1% |
| Värmdö | 93,3% | 90,4% | 85,8% | 81,4% |
| Österåker | 93,8% | 90,4% | 86,1% | 82,0% |
| Saknas | 42,7% | 60,8% | 50,9% | 46,0% |

## Hur vi rapporterar

Region Stockholm rapporterar om covid-läget på tisdagar och fredagar

Uppgifterna om antalet sjukhusvårdade är en ögonblicksbild från samma dag som rapporten publiceras.

På grund av kvalitetssäkring av uppgifterna om avlidna finns det en eftersläpning i inrapporteringen.

Ett viktigt mål med rapporten är att publicera uppgifter så snabbt som möjligt, både efterregistreringar och korrigeringar kan komma att ske.

## Vill du prenumerera på liknande nyheter?

Din e-postadress

### Välj kategori

Hälsa och vård

Aktivera prenumerationen

Se fler kategorier samt läs om hur vi hanterar dina personuppgifter på prenumerationssidan ›

Hitta rätt i vården (http://www.1177.se/Stockholm/)

Planera din resa (https://sl.se/)

Färdtjänst och sjukresor (https://fardtjansten.regionstockholm.se/)

Region Stockholm – för en jämlik, öppen, hållbar och konkurrenskraftig Stockholmsregion där invånarna ges likvärdiga livschanser och hög livskvalitet.

## Om webbplatsen

- Om webbplatsen (/om-webbplatsen/)
- Prenumerera på nyheter (/Prenumerationssida/)
- Om kakor (cookies) (/om-webbplatsen/cookies/)
- Tillgänglighetsredogörelse (/om-webbplatsen/tillganglighetsredogorelse-for-regionstockholm.se/)
- Lediga jobb (/jobb-1/Lediga-jobb/)

## Om Region Stockholm

**Telefon:** 08-123 100 00

**E-post:** kontakt@regionstockholm.se

Kontakta Region Stockholm (/kontakt/kontakta-region-stockholm/)

Anslagstavla (/politik/anslagstavla/)

## Följ oss i sociala medier

- Facebook
- LinkedIn
- Instagram
- Youtube
- Twitter

The Wayback Machine - https://web.archive.org/web/20211022163826/https://www.1177.se/en/Stockho...

1177 VÅRDGUIDEN

Besök 1177.se, logga in för personliga e-tjänster eller ring 1177 för sjukvårdsrådgivning.

# Getting tested for COVID-19

*Lämna prov för covid-19 - engelska*

A test is needed to find out whether you have COVID-19. The test is done using a sample you provide. Get tested if you have symptoms.

It is good to know whether it is COVID-19 that is making you sick. You can then make sure that you do not pass it on to other people.

Choose a region at the top of the page for more information about what applies where you live.

Du kan läsa texten på svenska här.

## When should I get tested?

Get tested if you have symptoms that could be caused by COVID-19. Here are some common symptoms:

- cough
- fever
- sore throat
- runny or stuffy nose
- loss of smell or taste
- headache
- muscle aches
- nausea
- stomach pain
- diarrhoea
- difficulty breathing
- tiredness.

Read more about the disease in the text called COVID-19.

You may also need to get tested if any of the following apply:

- You have been in contact with someone who has COVID-19.
- A doctor or nurse asks you to get tested.

Most people who have had COVID-19 are immune to it for at least six months afterwards.

Immune means protected against getting infected and sick again.

Get tested if you had COVID-19 more than six months ago and you get sick again.

You should also get tested if you are vaccinated and get sick. Read more in the text called Vaccination against COVID-19.

## You may need to get tested when travelling to Sweden

If you have been in another country, you may have to get tested when travelling to Sweden.

Read about the rules that apply when travelling to Sweden.

Stockholms län

## If you have recently been abroad

If you have been to a country outside the Nordic region, it is recommended that you test yourself after arrival and be careful with whom you meet the next week after the trip.

If you test positive for covid-19, your healthcare provider will inform you of the steps you must take in order to avoid the risk of infecting others.

## Order a test

The most common test is called a PCR test. You can order a PCR test.

Stockholms län

## Arranging for a test

1. Begin by downloading the *Alltid öppet* app from Appstore, Google Play or App Gallery to your telephone or your tablet.
2. You need *mobilt BankID* or *Freja eID Plus*.
3. Using the app, click on *Testing for covid-19*.
4. Answer a few questions on the telephone or tablet.
5. You will be given a code. Write it down.
6. Click on the link to 1177.se for ordering and scheduling.
7. Choose *Hemtest (home test)*, *Drive-in-test* or *Hämta/Lämna på apotek (pick-up/drop-off at a pharmacy)*. *Hemtest* means that a test kit will be sent to your home and you will do the test at home. *Drive-in-test* means you can take a car to a place where test kits are distributed. If you choose *Hämta/lämna på apotek* the person who picks-up / leaves the test kit must be free of symptoms.

It is important that you remain at home if you have symptoms even if you have not been able to arrange for a test.

## If you cannot schedule a test via the app or by telephone

If you don't have *mobilt BankId* or a personal ID number (*personnummer*), or if you have a protected or confidential ID (*skyddat id),* you can contact your healthcare centre and get help from them.

If you have symptoms of covid-19 and live close to a drop-in testing station, you can go there. There are now four units that visit 15 location in our county on weekdays. You will need an ID and a mobile number.

- Drop-in stations - locations and open hours (in Swedish)
- Instruction of how to take the test - other languages

# What about children?

All children aged at least 6 years can submit a test in this way. Younger children will not normally need to take a test.

**For children aged 6-12 years** you will need to act as your child's representative both when using the *Alltid öppet* app and on 1177.se. It will also be your responsibility to perform the test on your child.

This is what to do to become a representative in Alltid Öppet:

1. Click on "*Sök vård och provtagning för ditt barn*" in the app.
2. Click on "*Lägg till barn*" and add your child´s identity number. Then click on "*Sök vård och provtagning för ditt barn*" to switch to representative mode.
3. Select "*Provtagning för Covid-19*" and then "*Provtagning för Covid-19 på barn*".

N.B. When you then log into 1177.se, you need to change to representative mode there too. There is thus no link between the logins in Alltid öppet and 1177.se.

If your child requires a medical assessment, you can also contact your local healthcare centre (*vårdcentral*) and make an appointment for the child to be tested there.

**For children aged 13 or above,** they will need to obtain their own Mobilt BankID. You can also contact your health centre to be referred to a testing location where you can get tested.

### How will I receive the results?

The results usually come within 48 hours. You will receive a text message when your results are available and you log in to 1177.se to view them.

You can find answers to frequently asked questions about testing here (In Swedish).

## How testing works

A sample is taken with a special swab in your nose, your throat or both.

It may feel strange, but it does not hurt.

A nurse can collect the sample.

You can also collect the sample yourself. If this is the case, you will get more information on what you need to do.

## Stay at home until you get your results

It is important that you stay at home until you get your test results.

## If you have COVID-19

If the test results show that you have COVID-19, stay at home until you are no longer contagious. You are no longer contagious when all of the following apply:

- You are feeling a lot better.
- You have been fever-free for two days.
- At least seven days have passed since you became ill.

You do not need a new test to find out whether you are still contagious.

Anyone living with you may also need to stay home if you have COVID-19. You will get information about this when you get your test results.

Read more in the text called What can I do if I have COVID-19?

Stockholms län

During this time, you must:

- not go to work or school
- avoid meeting any people other than those with whom you live
- not go to shops or travel by public transport
- comply with special hygiene routines: be sure to wash your hands frequently, use a separate towel, and cough into your arm or into a tissue.
- notify staff of your infection when you seek medical care.
- read the rules you must adhere to in accordance with the Communicable Diseases Act.

## Do this immediately

If you have an ongoing covid-19 infection, you must also contribute to tracking the infection, in accordance with the provisions of the Communicable Diseases Act. The most important aspect is to inform anyone you may have infected. You must therefore do the following immediately:

1. Consider which people you have been in close contact with during the period when you were already contagious – i.e. from 48 hours before you first started feeling ill and experiencing symptoms of covid-19 until now. This applies to people you live with, people who have been visiting you, and those you have visited, i.e. friends, colleagues and health care professionals that do not already know about your infection.

   Close contact means having been together with a person for at least 15 minutes, less than 2 metres apart, also outdoors. People with whom you live are always considered to be close contacts.

2. Call, text or e-mail the people with whom you have been in close contact, and inform them that they may have been exposed to infection. You can find suggestions for what to write below.

3. Ask them to read the following information: Information for anyone who has come into contact with a person with covid-19 (in Swedish).

4. If they are in high school age and older; inform them that they should be tested immediately even if they have no symptoms. They should also meet as few people as possible until 14 days have passed since you met and work or study from home if they have the opportunity. If they get symptoms, they should stay home and be tested again. In case of symptoms, testing of children who go to pre-school class or primary school is also recommended.

5. If someone you live with works or studies, that person must work or study from home for at least 7 days. Children must also stay home from preschool/school for 7 days. See "Information for anyone who has come into contact with a person with covid-19 (in Swedish)". If you booked the test via the app Alltid öppet, you both can contact the Infection Tracking Team with questions via the app. Select "*Kontakta smittspårningsteamet för Covid-19*". If you took the test elsewhere, contact that care provider for further questions.

6. If you have met people at your workplace, school, training center or similar, and it´s possible that they may have been infected, but you for various reasons can not inform them yourself, get in contact with a manager or equivalent. He or she can help with infection tracing by passing on information. Ask him or her to share the following information (in Swedish)

7. If you booked the test via the app Alltid öppet and 1177.se, you´ll need to confirm that you have completed steps 1-6 above in the app. Go to "*Infection tracking of Covid 19*".

Please note that you may be contacted by phone by staff from Region Stockholm who work with infection-tracking.

**Suggestions for what to write**

*Hello! I have just found out that I have covid-19. I am contacting you as part of the work to track the infection. Since we have recently met, it is possible that you have been also exposed to infection, and Smittskydd Stockholm therefore recommends that you read the following information: 1177.se/Stockholm/info-covid-*

*kontakt (currently only available in Swedish). It is also important for you to get tested.*

### Do you have any questions?

If you wish to discuss your test results, you can call or chat with your healthcare centre (*vårdcentral*). If your symptoms become worse, you must contact 1177 Vårdguiden by telephone. In a life-threatening situation, call 112.

There is no reason to take a new test to confirm that you are no longer contagious. Test results may continue to be positive for a long time, even after you have ceased to be considered contagious.

### Information in other languages

Information for patients (issued by infectious disease specialists, including restrictions) has been translated into other languages. You can choose from ten different languages by clicking on the green button marked "*Svenska*" in the top right corner of the linked page.

#### Information and rules of conduct for household contacts

If you live with or have lived with someone who has covid-19, you may have been exposed to infection. This means there is a risk that you could become ill. To reduce the risk of infection spreading to others, you must follow certain rules of conduct.

You can choose from different languages by clicking on the green button marked "*Engelska*" in the top right corner of the linked page.

## If you do not have COVID-19

If the test results show that you do not have COVID-19, stay at home until you feel better and do not have a fever.

Stockholms län

#### Couldn´t your test be analysed or assessed?

If you recieve the answer that your test could not be analysed or assessed, the reason could be, for example, that the sample leaked, that something happened at the laboratory or that something went wrong technically which meant that the sample could not be analysed. If you wish to, you can then register for renewed testing after three days.

## Continue following the authorities' recommendations

When you are healthy, it is important that you continue reducing the risk of spreading the virus. This is true even if you are immune. Make sure you do the following:

- Wash your hands often.
- Keep your distance from other people.
- Follow the authorities' guidelines to reduce the spread of the virus.

## More information about COVID-19

Call 08-123 680 00 if you have any questions about COVID-19 and want to talk to someone who speaks English. The phone line is open Monday to Friday from 09:00 to 15:00.

READ MORE ABOUT COVID-19 IN ENGLISH

→ Collective information about COVID-19 - 1177.se

→ Public health agency of Sweden (Folkhälsomyndigheten) - COVID-19 FAQ in English

→ Public health agency of Sweden (Folkhälsomyndigheten) - Recommendation to reduce the spread of COVID-19

→ Public health agency of Sweden (Folkhälsomyndigheten) - Recommendation for those travelling or who have travelled to Sweden from the United Kingdom South Africa

---

**Revision date:**

2021-07-14

**Editor:**

Ida Friedmann, Nationella redaktionen 1177.se

**Reviewer:**

Jerker Jonsson, Doctor, specialist in infectious diseases, Stockholm

Region Örebro län



# Provtagning, covid-19

I Örebro län kan du ta ett prov för att se om du har covid-19. Provet kallas PCR-test och visar om du har en pågående infektion av covid-19.

## Covid-19 och resor

Det kan vara olika rekommendationer och regler som gäller kring provtagning för covid-19 och intyg beroende på vart du vill åka eller var du befunnit dig när du reser in i Sverige. Du är själv ansvarig för att ta reda på vad som gäller för ditt resmål och de länder du reser igenom på vägen dit.

Läs mer information om:

- resor och covidbevis via 1177.se
- rekommendationer för resenärer på Folkhälsomyndighetens webbplats

## Hämta testkit för covid-19 från lådor – utan att boka tid

Runt om i länet finns särskilda lådor där du som behöver testa dig för covid-19 kan hämta ett testkit och lämna in ditt prov när du tagit det. Du behöver inte beställa prov eller boka tid. Däremot behöver du ha e-legitimation för att kunna koppla ditt prov till dig.

Läs mer om lådorna och deras öppettider på 1177.se

Om du skanar e-legitimation ringer du din vårdcentral för tidsbokning.

## Mer information

→ Lämna prov och få provsvar om covid-19, 1177.se

→ Så lämnar du prov för covid-19 i Örebro län, 1177.se

Senast uppdaterad: den 27 augusti 2021

The Wayback Machine - https://web.archive.org/web/20211018044604/https://www.1177.se/Orebrolan/...

1177 VÅRDGUIDEN

Besök 1177.se, logga in för personliga e-tjänster eller ring 1177 för sjukvårdsrådgivning.

Innehållet gäller Örebro län

# Provtagning för covid-19 (PCR) i Örebro län

Här finns information om hur provtagning för covid-19 går till i Örebro län.

Läs om provtagning för covid-19 på lätt svenska.
Read about covid-19 and getting tested in other languages.

Här kan du läsa mer om när och vem som ska lämna prov för covid-19.

**Ring inte 1177 om prov för covid-19**

**1177 Vårdguiden på telefon kan inte hjälpa dig med provsvar eller att boka, omboka eller avboka ett prov för covid-19.**

## För dig med e-legitimation: hämta testkit från låda

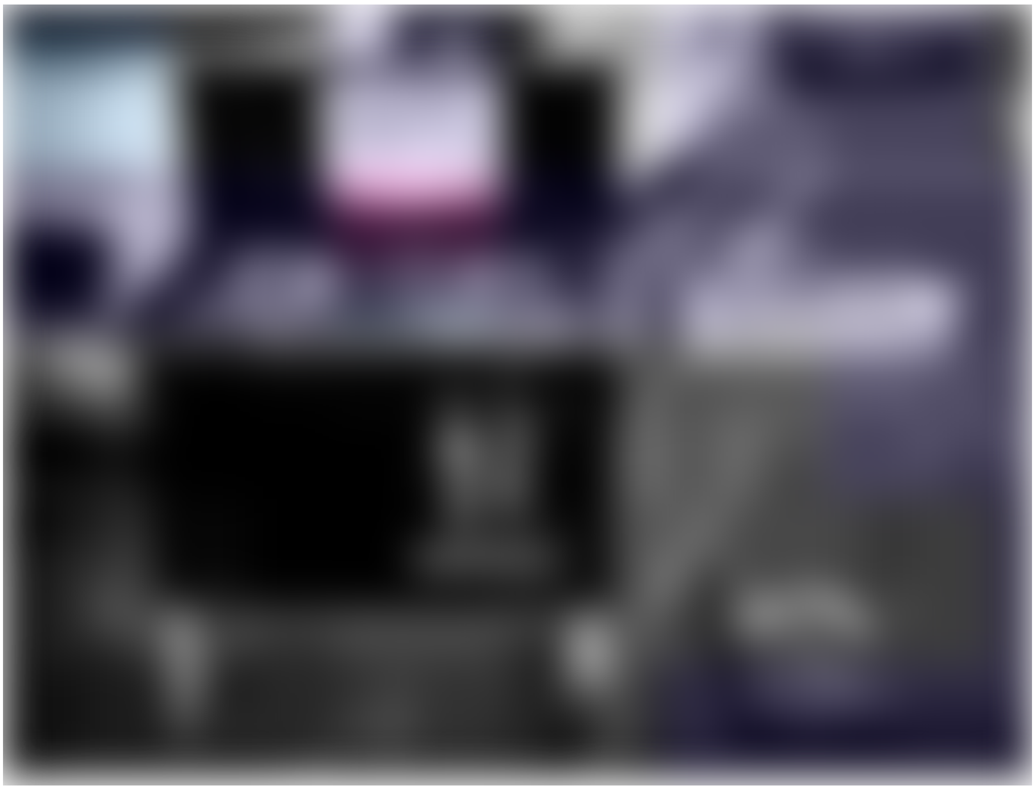

Låda med testkit för covid-19.

Du som behöver testa dig för covid-19 kan hämta testkit och lämna in ditt prov via särskilda lådor som finns runt om i länet. Du behöver ha e-legitimation för att kunna registrera ditt prov på 1177.se.

### Så här går det till:

- Hämta testkit från någon av lådorna
- Testa dig
- Registrera prov på 1177.se med hjälp av e-legitimation
- Lämna in prov i någon av lådorna för analys
- Provsvar kan du se inom 3 dygn genom att logga in på 1177.se
  (godkänn aviseringar under inställningar i inloggat läge, så får du en avisering via sms eller e-post när provsvaret kommit).

Det går bra att hämta testkit till andra, till exempel dina familjemedlemmar.

Om möjligt hämtas och lämnas testkitet av någon som är symtomfri. Om det inte är möjligt är det viktigt att du tar dig till lådan på ett smittsäkert sätt. Var noggrann med handhygien och använd gärna munskydd för att minska risken att smitta andra.

## Så testar du dig och registrerar ditt testkit från låda

För att testa dig med ett testkit från en låda behöver du ha e-legitimation (bankID eller Freja eID Plus). Med hjälp av e-legitimationen loggar du in på 1177.se och kopplar ditt prov till dig.

Det är viktigt att varje prov registreras på den person som tagit provet.

#### Barn och unga

Prov från barn till och med 12 år registreras av vårdnadshavare. Som vårdnadshavare loggar du in på 1177.se, aktiverar ombudsläget för ditt barn och registrerar provet på barnet.

Barn från 13 år registrerar sitt prov med egen e-legitimation. Vårdnadshavare kan inte agera ombud för barn från 13 år.

Läs mer om e-legitimation och hur du skaffar e-legitimation.

### Instruktioner

- Testa dig för covid-19 med testkit från låda (pdf)
  Instruktioner med QR-kod/testkitsnummer som krävs för att registrera provet på 1177.se följer med varje testkit.
- Bildguide: testa dig för covid-19 med testkit från låda och registrera **ditt** test (pdf)
- Bildguide: registrera **barns** prov på 1177.se **som ombud** (pdf)

Video: //play.mediaflowpro.com/ovp/16/87BB2EBV13

Filmen visar hur du testar dig för covid-19 med testkit från Lådan.

## Frågor och svar om testkit via låda

### Tänk om jag gör fel när jag ska ta prov och inte har någon att fråga?

Provtagningsmetoden, som består av en kombination av prov från svalg och näsa, är samma metod som Region Örebro län använt i snart ett år med bra erfarenhet. Den är utformad för att ge ett så säkert svar som möjligt vid egenprovtagning.

Du behöver inte vara orolig för att näspinnen inte kommer tillräckligt långt in. Kombinationen med gurgelprovet gör att det blir ett bra prov. Men det är viktigt att följa instruktionerna du får. Läs igenom hela instruktionen innan du börjar. Du kan också titta på vår instruktionsfilm innan du testar dig.

### Hur vet jag om jag registrerat mitt prov på rätt sätt?

Det är viktigt att slutföra sin provregistrering när du loggat in på 1177.se. En grön ruta med informationen ”Testkit registrerat!” betyder att du är klar. Slutför du inte provregistreringen får du inget provsvar. Om du blir osäker i efterhand om du slutfört registreringen kan man som inloggad på 1177.se se om det finns en beställning under Egen provhantering, läs mer här.

### Vad ska jag göra om min beställning - mitt prov som jag trott att jag registrerat - inte syns i ”Egen provhantering” på 1177.se?

**Om du har kvar instruktionerna med testkitsnumret** kan du registrera ditt prov på nytt. Din beställning tas då omhand på laboratoriet där ditt prov sparas ett par dagar och du kommer att få provsvar. Du registrerar ditt prov enligt instruktionerna. En grön ruta med informationen ”Testkit registrerat!” på 1177.se betyder att du slutfört registreringen och är klar.

**Om du inte har kvar testkitsnumret** behöver du tyvärr hämta ett nytt testkit och börja om. Alternativt beställa prov och boka en tid på någon av våra provtagningsmottagningar.

### Kan jag registrera hela familjens prov på mig?

Nej. Varje prov måste registreras på den person som provtagits. För barn från förskoleklass (5-6 år) till och med 12 år behöver du som vårdnadshavare registrera barnets testkitsnummer i ombudsläge. Information finns i instruktionerna.

### Kan jag testa mig vid lådan?

Undvik att testa dig precis intill lådan eller på platser där kö eller trängsel kan uppstå. Om du kör till lådan är bilen en bra plats att provta sig på, då kan du snabbt lämna in provet efter själva provtagningen.

Vi ber dig att **inte** gå in i våra verksamheter intill lådan, för att eventuell smitta inte ska spridas vidare.

## Kan jag hämta testkit från en låda och lämna in prov i en annan låda?

Ja, det går bra att lämna in prov i en annan låda än den du hämtade testkit från. Här finns lådornas öppettider.

Däremot ska du **inte** gå in på vårdcentral för att lämna in prov.

## Här finns lådor

På vardagar finns en låda utanför samtliga vårdcentraler i Örebro län under dagtid. På kvällar och helger finns en låda utanför vårdcentralernas jourmottagning i varje länsdel. Samtliga lådor står utomhus, följ skyltning.

### Öppettider vardagar dagtid

- **Lådor utanför länets samtliga vårdcentraler**
  **måndag - torsdag 08.00-16.30**
  **fredagar samt dag innan röd dag 08.00-15.45**

  Obs! I Kumla finns lådan utanför vårdcentralens Fylstamottagning. Lådan är öppen 08.00-21.00 under vardagar.

### Öppettider kvällar och helger

- **Örebro**
  **Utanför vårdcentralernas jourmottagning**
  **måndag-fredag klockan 16.00-21.00**
  **lördag-söndag 08.00-21.00**
  Besöksadress: Nygatan 7
  Karta till vårdcentralernas jourmottagning, Örebro
- **Norra länsdelen**
  **Utanför Lindesbergs vårdcentral**
  **Lådan finns tillgänglig dygnet runt**
  Besöksadress: Banvägen 24
  Karta till Lindesbergs vårdcentral
- **Södra länsdelen**
  **Utanför Kumla vårdcentral Fylstamottagningen/jourmottagningen**
  **Måndag-fredag klockan 08.00-21.00**
  **Lördag-söndag klockan 12.00-21.00**
  Besöksadress: Sörbyvägen 8
  Karta till Kumla vårdcentral Fylstamottagningen/jourmottagningen
- **Västra länsdelen**
  **Utanför Baggängens vårdcentral/jourmottagningen**
  **Lådan finns tillgänglig dygnet runt**
  Besöksadress: Baggängsvägen 59
  Karta till Baggängens vårdcentral/jourmottagningen

## För dig utan e-legitimation: ring och boka tid

Du som saknar e-legitimation eller svenskt personnummer kan ringa och boka tid för egenprovtagning (du tar testet själv enligt instruktioner) eller upphämtning av ett testkit på vårdcentral/vårdcentralsjour som du sedan lämnar in i någon av regionens lådor för covid-test. Du behöver legitimera dig på plats. Om du skickar ett ombud för upphämtning av testkit måste hen visa bådas legitimation.

Om du ingår i smittspårning eller varit utomlands och har uppmanats att testa dig vid två tillfällen kan du få med dig ett testkit för test nummer två, och ta det andra

testet hemma på egen hand. Testet lämnas sedan in i någon av regionens lådor för covid-test.

### Vardagar

Ring din vårdcentral.

### Helger

Ring 019-602 88 95
Telefontid lördag, söndag och helgdag klockan 09.00-12.00.

Bokar du tid under helgen kommer du att hänvisas till vårdcentralsjouren i din länsdel. Hitta till vårdcentralsjouren i:

- Örebro
- Norra länsdelen
- Södra länsdelen
- Västra länsdelen

Obs! Telefonen bemannas av sekreterare som inte kan erbjuda medicinsk bedömning.

### Så här tar du PCR-test

Video: //play.mediaflowpro.com/ovp/16/87BBVCQUV5
Filmen finns textad på arabiska, engelska och somaliska samt teckentolkad. Välj språk via språkikon. You can select different subtitle languages by clicking on the subtitle icon on a video.

## Mer information

Läs mer på 1177.se:

- Om covid-19
- Lämna prov för covid-19
- Ditt provsvar för covid-19
- Till dig som haft nära kontakt med person som har covid-19
- För barn: om corona och covid-19
- Testning i samband med resa (Covid-19: resor och covidbevis)

---

**Senast uppdaterad:**

2021-10-15

**Redaktör:**

Lisa Lilja, 1177 Vårdguiden, Region Örebro län

**Granskare:**

Lena Adolfsson, biträdande hälso- och sjukvårdsdirektör, Region Örebro Län

The Wayback Machine - https://web.archive.org/web/20210920053155/https://www.1177.se/Dalarna/sju...

1177 VÅRDGUIDEN

Besök 1177.se, logga in för personliga e-tjänster eller ring 1177 för sjukvårdsrådgivning.

Innehållet gäller Dalarna

# Självprovtagning covid-19 i Dalarna

Provet kan visa om du har covid-19 just nu. Du tar provet själv vid en av våra provtagningsstationer eller hämtar ett provtagningspaket på ett av våra utvalda apotek.

Självprovtagning kan endast bokas genom att logga in på 1177 Vårdguidens e-tjänster.

BOKA SJÄLVPROVTAGNING

**Covid-19: Så här provtar du dig själv - film**

Video: //play.mediaflowpro.com/ovp/16/32BBKCZ485

## Självprovtagning covid-19

Covid-19 påminner ofta om vanlig förkylning eller influensa. Läs mer om symtom.

Vid symtom ska du boka in tid för provtagning. Du kan bara lämna ett prov per sjukdomsperiod.

Särskilda rekommendationer gäller om du reser in i Sverige.

Provet kostar inget.

### Vuxna och ungdomar från 13 år

Du bör boka tid för självprovtagning covid-19 (PCR-test) i Dalarna om följande stämmer in på dig:

- Du har symtom på covid-19
- Du har haft symtom i minst 24 timmar
- Du är så kallad hushållskontakt och har informerats om att du ska ta prov
- Du har av smittspårare uppmanats att ta prov
- Om du varit utomlands

Du kan ta provet på dig själv i näsa och svalg med provtagningspinne. Förälder/vårdnadshavare ska hjälpa ungdomar under 16 år att ta provet.

### Barn 10-12 år

Förälder eller annan vårdnadshavare rekommenderas att boka tid för självprovtagning om:

- Barnet har symtom på covid-19
- Barnet har haft symtom i minst 24 timmar
- Barnet är så kallad hushållskontakt och har informerats om att provtagning ska göras
- Barnet har av smittspårare uppmanats att ta prov

Förälder/vårdnadshavare ska hjälpa barnet att ta provet.

Covid-19 påminner ofta om vanlig förkylning eller influensa. Läs mer om symtom.

## Så här bokar du

### Du som är 13 år eller äldre och boende i Dalarna

1. Du loggar in på 1177 Vårdguidens e-tjänster med Mobilt BankID, BankID, Freja e-id plus eller annan e-legitimation. Obs! Bankernas SäkerhetsID för ungdomar fungerar inte.
   Så här skaffar du e-legitimation om du är 13-17 år
2. Du beställer provtagning under rubriken "Regionen rekommenderar" - "Självprovtagning covid-19 för dig som är sjuk".
3. Du väljer provtagningsplats.
4. Du bokar tid.
5. Skriv in din e-postadress för att få bekräftelse på din bokning och för att kunna av- och omboka din tid.
6. Nu är det klart i 1177 Vårdguidens e-tjänster.

BOKA SJÄLVPROVTAGNING

### Barn 10-12 år som är boende i Dalarna

Förälder eller annan vårdnadshavare loggar in i 1177 Vårdguidens e-tjänster och beställer prov via ombudsläge, samt hjälper barnet att utföra provet.

Längre ner på sidan finns en film som visar hur du som ombud bokar för ditt barn via e-tjänsterna. Du kan även läsa mer på sidan Gör ditt barns vårdärenden via nätet.

### Barn 6-9 år och övriga grupper som är boende i Dalarna

Förälder eller annan vårdnadshavare tar kontakt med vårdcentralen och bokar tid för provtagning för covid-19. Provet utförs på vårdcentral. Provsvar meddelas via brev, kontakt med vården eller 1177 Vårdguiden e-tjänster. Gäller grupperna nedan:

- Barn från förskoleklass till 9 år
- Särskilt boende (SÄBO)
- Personer kontaktade vid smittspårning
- Personer utan personnummer

### Du som är på besök i Dalarna

Läs mer om hur du som besöker Dalarna gör för att boka tid för självprovtagning covid-19.

### Support

**Om du har frågor om hur du bokar tid** för självprovtagning och behöver support kan du skicka e-post till provtagning.covid@regiondalarna.se. Just nu får vi många frågor och vi återkommer till dig så snart vi har möjlighet.

**Support på telefon:** ring 010-249 92 88
Öppettider: måndag-fredag 08:00-12.00 och 13.00-16:00

Du kan ringa detta nummer för att få svar på dina frågor eller vägledning hur du bokar tid. Personer utan e-legitimation kan också få hjälp att boka via supporten.

## Provtagning av personal

Från den 17 december gäller självprovtagning för all vård- och omsorgspersonal i region och kommun som inte arbetar patientnära. Mer information på Region Dalarnas webbplats:

Provtagning av personal inom hälso- och sjukvård samt vård och omsorg

**Så här bokar du - film**

Video: //play.mediaflowpro.com/ovp/16/31BBI7BJRP

**Så här av- och ombokar du - film**

Video: //play.mediaflowpro.com/ovp/16/20BBM7YJDP

**Så här beställer du som vårdnadshavare tid för självprovtagning för covid-19 till barn - film**

Video: //play.mediaflowpro.com/ovp/16/85BBU74J0P

## Boka din självprovtagning här

### Provtagningsstationer och apotek

Här kan du se på vilka orter de mobila provtagningsstationerna finns och vilka apotek som tillhandahåller tjänsten.

Du tar dig till provtagningsstationen med bil. Till vissa stationer kan du även gå eller cykla, se listan nedan. Från vecka 28 gäller enbart gå eller cykla till provtagningsstationerna i Hedemora, Malung och Säter.

Provtagningsstationer för självprovtagning finns på följande orter:

- Avesta, ishallen
- Borlänge, gamla Folkets Park (OBS! Ej Galaxen hotell). **Även gående och cyklande. Även öppet på lördagar.**
- Borlänge, provtagningsbuss vid Vårdcentral Jakobsgårdarna, Jaxtorget 7 (baksidan av vårdcentralen) måndagar, onsdagar och fredagar
- Borlänge, provtagningsbuss vid Vårdcentral Kvarnsveden, baksidan av vårdcentralen (korsningen Sveaborgsgatan-Risbindargatan), tisdagar och torsdagar
- Falun, Lugnet, stora grusplanen. **Även gående och cyklande. Även öppet på lördagar.**
- Gagnef, Centrumvägen
- Hedemora, Folkets park. **Även gående och cyklande.**
- Idre, Smijolet IP
- Leksand, Kulturhuset. **Även gående och cyklande.**
- Ludvika, Hillängens IP
- Malung, Södra hantverkargatan. **Även gående och cyklande.**
- Mora, Cirkusplatsen Tingnäs. **Även gående och cyklande. Även öppet på lördagar.**
- Orsa, Ishallen
- Rättvik, Reparatörvägen 10
- Smedjebacken, Frejgatan 27
- Sälen by, Sälenhallen
- Säter, Folkets hus. **Även gående och cyklande.**
- Vansbro, vid Medborgarhuset
- Älvdalen, simhallen

Följande apotek tillhandahåller tjänsten:

- Avesta, Apoteket Kronan
- Borlänge, Apoteket Falken
- Borlänge, Apoteket Kupolen
- Djurås, Apoteket Djurås
- Falun, Apoteket Engelbrekt
- Falun, Apoteket Falu lasarett EA
- Leksand, Apoteket Leksand
- Ludvika, Apoteket Liljan
- Mora, Apoteket Bävern EA
- Svärdsjö, Apoteket Kråkan
- Vansbro, Apoteket Tallen
- Älvdalen, Apoteket Björnen

## Så går provtagningen till

### På provtagningsstation:

1. När det är dags för din tid tar du dig till den provtagningsplats du valt med bil. Du får inte åka kollektivt, ta taxi, cykla eller gå eftersom du har symtom.
2. På provtagningsplatsen ska du stanna där du blir anvisad av personal eller skyltar. Stanna i bilen.
3. Du måste ha med dig giltig fotolegitimation. Det är också bra att ha med handsprit, pappersnäsdukar, en liten påse för skräp och en liten spegel om du har en.
4. Personalen kommer att gå runt och ge dig provtagningspaketet genom din öppna bilruta. Följ instruktionen som finns i provtagningspaketet.
5. När du genomfört provtagningen stannar du där du är så kommer personalen och hämtar provet.
6. Du får uppvisa legitimation.

**På apotek:**

**Obs!** För att hämta provtagningspaket på ett apotek behöver du använda dig av ett ombud. Ombudet ska inte ha några symtom när hen hämtar paketet och hen ska ha med sig din och sin egen legitimation.

Provet skall förvaras i kyla efter provtagning och lämnas till samma apotek där det hämtades ut, snarast möjligt.

Observera att sista tiden för inlämning av prov inte överensstämmer med apotekets vanliga öppettider.

Du ska ha bokat tiden själv innan. Tänk på att provet ska lämnas in samma dag som provtagningen genomförs.

## Så får du provsvar

1. Inom 1-3 dygn får du provsvar i 1177 Vårdguidens e-tjänster. Det gäller alla som genomfört självprovtagning oavsett ålder. Vill du veta när provsvaret kommit? Kontrollera att du ställt in att du vill få avisering via sms eller e-post när du bokar tiden inne i 1177 Vårdguidens e-tjänster. Missade du det kan du när som helst logga in och ställa in önskan om aviseringar under inställningar längst upp. Det är bra för all kommunikation via 1177 Vårdguidens e-tjänster.
2. Medan du väntar på provsvaret ska du hålla dig hemma. Blir du sämre ring 1177 för råd eller kontakta vårdcentralen genom att skicka meddelande via 1177 Vårdguidens e-tjänster eller ring. Vid akut sjukdom ring 112.
3. Om du inte fått ditt provsvar efter fyra dygn. Logga in på 1177.se om du inte fått provsvar kanske har fått provsvar i inkorgen under meddelanden ändå. Att du inte fått något provsvar kan bero på flera saker. Det kan vara att många har symtom och provtar sig nu vilket gör att det kan ta längre tid än vanligt. Ibland kan laboratoriet behöva göra om analysen av provet, vilket också gör att det kan ta längre tid att få svar. Om det gått mer än 4 vardagar sedan du provtog dig så boka ett nytt test.
4. **Ring inte 1177 eller vårdcentralen om ditt provsvar** - de har ingen information att ge om provsvar för prover tagna på självprovtagningen.

**Logga in på 1177 Vårdguidens e-tjänster och ställ in dina aviseringar - film**

Video: //play.mediaflowpro.com/ovp/16/83BB87RJXP

## Så tolkar du provsvaret

Provsvaret kommer vara ett av följande tre alternativ:

1. **Virus påvisat**, det betyder att du har covid-19.
2. **Virus ej påvisat**, betyder att du inte har covid-19.
3. **Prov ej bedömbart**, det betyder att du ska boka tid för ny provtagning.

## Positivt provsvar för covid-19

Är ditt provsvar positivt har du en pågående infektion och det är viktigt att du stannar hemma för att inte riskera att smitta andra. Du måste också följa smittskyddslagen.

När ditt provsvar visar att du har en pågående infektion orsakad av covid-19 ska du vara hemma i minst sju dagar från att du fick symtom, samt tills du varit feberfri under två dygn och känner dig allmänt bättre. Då bedöms du inte längre vara smittsam.

Du behöver inte ta ett nytt prov för att se om du är smittfri eller av annat skäl. Provet kan vara positivt lång tid utan att du för den skull bedöms vara smittsam.

### När du har en pågående infektion ska du:

- inte gå till arbete eller skola
- undvika att träffa andra personer än dem du bor med
- inte handla i butiker, på apotek eller resa med allmänna transportmedel
- följa särskilda hygienrutiner: vara noggrann med att tvätta händerna ofta, använda egen handduk och hosta i armvecket eller i näsduk.
- informera om att du är smittad om du söker vård.
- ta del av de gällande förhållningsregler Covid-19: Patientinformation (finns också på andra språk än svenska)

## Smittspårning - gör så här

Vem som helst som har tagit ett positivt covid-prov kan ringas upp av smittspårare från Region Dalarna. Alla blir inte uppringda, men många. Samtal från smittspårningsläkare visas då som hemligt eller dolt nummer.

Enligt smittskyddslagen ska du medverka i smittspårning om ditt provsvar är positivt. Det viktigaste är att informera personer som du kan ha smittat.

**Gör så här:**

**1.** Tänk igenom vilka personer du har varit i nära kontakt med under den tid period du hittills varit smittsam (från och med 48 timmar innan du först kände dig sjuk/fick symtom tills nu).

Med nära kontakt menas en person som du träffat under minst 15 minuter inom 2 meters avstånd. Personer du bor ihop med räknas alltid som nära kontakter. Gå igenom enligt följande:

- personer du bor med, personer som varit på besök hos dig, personer du har besökt under den aktuella tiden
- vänner som du har umgåtts med nära och arbetskamrater som du haft nära kontakt med
- vårdpersonal – om du sökt vård och de inte redan känner till din covid-19-infektion.

**2**. Ring, sms:a eller mejla dem du varit i nära kontakt med och informera om att de kan ha blivit utsatta för smitta. Den här informationen kan du använda: Till dig som har utsatts för coronasmitta

**3.** Har du träffat personer på din arbetsplats eller inom annan verksamhet (skola, träning, fritidsverksamhet) som du kan ha utsatt för smitta, men som du av olika anledningar inte själv kan eller bör informera, tar du hjälp av din chef/arbetsledare, lagledare, rektor eller motsvarande. Hen kan informera vidare och hjälpa till med smittspårningen.

Är du så sjuk att du inte orkar eller av andra skäl inte kan informera enligt ovan kontakta du Region Dalarnas Smittskyddsenhet, e-post: smittskydd.dalarna@regiondalarna.se

Om personer du bor med arbetar eller går i skola, ska de arbeta/studera hemifrån minst 7 dagar och följa förhållningsreglerna för hushållskontakter. Den som är vaccinerad med sin andra dos vaccin för minst två veckor sedan, behöver inte vara hemma om den är hushållskontakt, så länge den är frisk.

De kan även få så kallad smittbärarpenning.

## Hushållskontakter och smittbärarpenning

En **hushållskontakt** är någon som bor eller har bott under samma tak som en covidsjuk person, under den tid hen har varit smittsam, det vill säga från 48 timmar innan hen blev sjuk till och med 7 dagar efter insjuknandet.

Hushållskontakter som arbetar ska i första hand **arbeta hemifrån**. Skolelever ska studera hemma (som hushållskontakt räknas nu även barn under 16 år).

**Om du inte kan arbeta hemifrån kan du ansöka om smittbärarpenning** hos Försäkringskassan. Du behöver då ett läkarintyg. För att få ett läkarintyg ska du logga in på 1177 Vårdguidens e-tjänster och välja din vårdcentral.

1177.se E-tjänster: Allmän inloggning

Under mottagningens e-tjänster hittar du ”Ansökan om läkarintyg för smittbärarpenning för hushållskontakter till person med covid-19”. En e-legitimation, antingen BankID eller Freja eID Plus, behövs. Om du inte har e-legitimation ska du ringa din vårdcentral.

De uppgifter du behöver lämna i ansökan är:

- hur många timmar du arbetar per vecka
- datumet för när den covidsjuka i familjen tog sitt prov.

**Hushållskontakter från 10 års ålder och uppåt** ska från och med 18 januari ta prov 5 dagar efter det tillfälle den först smittade blev provtagen. Om provet är negativt och inga symtom utvecklas, kan hushållskontakten återgå till arbete, skola eller förskola sju dagar efter den sjuka i familjen tog provet.

Folkhälsomyndigheten har tagit bort möjligheten för vård- och omsorgspersonal som är hushållskontakter att ta PCR-prov redan efter 2-3 dagar för att återkomma tidigare i arbete vid negativt prov. Vård- och omsorgspersonal ska som alla andra, ta prov efter 5 dagar och återgå i arbete vid negativt provsvar.

Om du blir sjuk dessförinnan ska du boka tid för provtagning tidigare.

## Frågor och svar

Har du funderingar kring självprovtagningen kan du läsa mer i frågor och svar.

## Spara ditt prov i biobank

Ditt prov kan komma att sparas i biobank. Här kan du läsa mer om varför prov sparas i biobank.

Om du inte vill att ditt prov ska sparas i biobank behöver du fylla i och skicka in en nej-talong och skicka den tillsammans med proven till laboratoriet. Nej-talong hittar du här.

---

**Senast uppdaterad:**

2021-09-16

**Redaktör:**

Marie Jakobsson, Region Dalarna

**Granskare:**

Helena Ernlund, bitr. smittskyddsläkare, Smittskyddsenheten Falu lasarett, Falun
